# Molecular monitoring and multiplex qPCR-based detection of *Cydia pomonella* and *Grapholita molesta* in apple orchards of Kazakhstan

**DOI:** 10.3389/finsc.2026.1897268

**Published:** 2026-07-31

**Authors:** Alibek Makhambetov, Valeriya Kostyukova, Bakyt Dulat, Zarina Dairbekova, Gulnur Zholdasbek, Kairova Gulshariya, Marina Khusnitdinova, Abay Sagitov, Dilyara Gritsenko

**Affiliations:** 1 Laboratory of Molecular Biology, Institute of Plant Biology and Biotechnology, Almaty, Kazakhstan; 2 Department of Molecular Biology and Genetics, Al Farabi Kazakh National University, Almaty, Kazakhstan; 3 Research Center AgriBioTech, Almaty, Kazakhstan; 4 Kazakh National Agrarian Research University, Almaty, Kazakhstan

**Keywords:** apple orchards, *Cydia pomonella*, *Grapholita molesta*, molecular identification, multiplex qPCR, phytosanitary monitoring, sequencing

## Abstract

**Introduction:**

Apple production in Kazakhstan is highly vulnerable to damage caused by fruit-infesting tortricid pests, particularly codling moths (Lepidoptera: Tortricidae). Accurate and rapid species identification is essential for effective pest management and phytosanitary monitoring in apple orchards.

**Methods:**

Field surveys were conducted in apple orchards of southern and southeastern Kazakhstan to investigate the codling moth complex. Collected specimens were identified morphologically and confirmed by sequencing a mitochondrial cytochrome c oxidase subunit I (COI) region. Comparative COI sequence analysis was used to develop a multiplex quantitative polymerase chain reaction assay employing Minor Groove Binder hydrolysis probes for simultaneous detection and differentiation of *Cydia pomonella* and *Grapholita molesta*.

**Results:**

Field surveys identified *C. pomonella* and *G. molesta*, with *C. pomonella* accounting for 83.3% of collected specimens. COI sequencing enabled reliable discrimination between the two species. The multiplex assay demonstrated high specificity, produced no false-positive amplification in non-target species, and achieved 100% detection down to 5 pg DNA per reaction.

**Discussion:**

The developed COI-based multiplex quantitative polymerase chain reaction assay provides a rapid, sensitive, and reliable tool for simultaneous identification of *C. pomonella* and *G. molesta*. It is suitable for phytosanitary monitoring, early detection, and management of quarantine-relevant tortricid pests in Kazakhstan.

## 1 Introduction

Apple production represents one of the most important sectors of horticulture in Kazakhstan and has both economic and ecological significance for the country. Kazakhstan is recognized as a center of origin of the cultivated apple, with native populations of *Malus sieversii* that form a valuable genetic reservoir for breeding and biodiversity conservation (1–4). The country’s apple orchards are concentrated predominantly in the southern and southeastern regions, particularly in Almaty, Zhetysu, Zhambyl, and Turkestan, where favorable climatic conditions support intensive commercial fruit production. In recent years, the total orchard area in Kazakhstan has approached 29,000 ha, while national apple production has increased due to the expansion of intensive orcharding systems and state-supported horticultural development programs.

The concentration of large orchard areas in southeastern Kazakhstan creates favorable ecological conditions for the development and persistence of phytophagous insects associated with fruit crops. Among them, tortricid fruit moths (Lepidoptera: Tortricidae), including *Cydia pomonella* L. and *Grapholita molesta* (Busck), are regarded as among the most economically important pests of pome fruit orchards worldwide because larval feeding directly injures fruits, resulting in yield reduction, premature fruit drop, and deterioration in marketable fruit quality (5–8). Particularly important among tortricid pests are *C. pomonella* (9, 10) and *G. molesta* (9, 11, 12), both of which are recognized as major pests of apple and other fruit crops throughout Eurasian fruit-growing regions, causing substantial economic losses in commercial orchards. *C. pomonella* has historically been regarded as the dominant pest of apple orchards worldwide (9, 10), whereas *G. molesta* has increasingly emerged as an important invasive and quarantine-relevant species in many temperate fruit production systems (12, 13).

Fruit infestation by both species leads to tissue destruction, frass contamination, premature fruit drop, deformation, and increased susceptibility to secondary fungal and bacterial infections (9). As a result, damaged fruits lose market value and marketability, causing significant economic losses in commercial orchards. Under conditions of high infestation and inadequate pest control, losses can be substantial and may reach near-complete crop failure in untreated orchards (9, 10). These pests, therefore, remain major targets of phytosanitary surveillance and integrated pest management programs in commercial fruit production.

Although *C. pomonella* remains the primary target of pest monitoring in apple orchards, recent studies indicate increasing complexity in tortricid communities due to climate change, intensification of fruit production, expansion of trade networks, and movement of planting material between regions (10, 13). These factors facilitate range expansion and the establishment of additional tortricid species that can occupy overlapping ecological niches. Of particular concern is *G. molesta*, a species native to East Asia that is now widely distributed across Europe, Asia, North and South America, and Oceania (13). Its successful spread is associated with multivoltine development, a wide host range, ecological plasticity, and rapid development of insecticide resistance.

In Kazakhstan, the phytosanitary significance of *G. molesta* has increased in recent decades. According to the European and Mediterranean Plant Protection Organization Global Database, the species was first recorded in Kazakhstan in 1985 and is currently classified as present with restricted distribution (*Grapholita molesta* (LASPMO)[Overview]| EPPO Global Database, n.d.). As of the most recent official assessment, *G. molesta* has been reported from three regions with a documented infested area of 587 ha. The species is officially included on the list of quarantine pests regulated in the Republic of Kazakhstan, underscoring its national phytosanitary importance. In addition, official phytosanitary inspection reports repeatedly document interceptions of *G. molesta* in imported consignments of apples, pears, and peaches entering Kazakhstan through regional trade routes, indicating continued risk of introduction and secondary spread (*Grapholita molesta* (LASPMO)[Overview]| EPPO Global Database, n.d.).

Despite the economic importance of fruit moths in Kazakhstan, molecular data on tortricid diversity and distribution in commercial apple orchards remain limited. Pest monitoring in orchards is still largely based on pheromone trapping and morphological identification. While adult specimens can often be distinguished morphologically, larval identification is considerably more challenging due to overlap in diagnostic characters among closely related taxa. Moreover, damage caused by larvae of different tortricid species within fruits is often visually indistinguishable under field conditions, complicating accurate diagnosis during routine phytosanitary surveys. This creates a significant need for rapid, reliable molecular diagnostic tools for species-level identification in both monitoring and quarantine applications.

Molecular identification based on mitochondrial DNA markers has become a standard approach for taxonomic resolution of Lepidoptera pests (14–17). DNA barcoding using COI region is widely applied for accurate identification of tortricid species and enables discrimination of morphologically similar taxa, including larval stages and damaged material (15, 18). To date, molecular diagnostic approaches for the identification of *C. pomonella* and *G. molesta* have mainly relied on conventional PCR (19) and PCR–RFLP (20). Conventional PCR-based methods remain widely used for species confirmation; however, they often require post-amplification processing such as gel electrophoresis, restriction digestion, or sequencing, which increases analysis time and limits their suitability for high-throughput routine screening. A real-time PCR assay for *C. pomonella* is currently available (21). However, the development of multiplex qPCR assays based on hydrolysis probes that enable the simultaneous detection and differentiation of *C. pomonella* and *G. molesta* using the COI marker within a single reaction remains an important objective for phytosanitary diagnostics and monitoring.

In the present study, we conducted large-scale field monitoring of tortricid fruit moths in apple orchards across southern and southeastern Kazakhstan between 2024 and 2026. We combined morphological examination with COI-based molecular identification to characterize species composition and confirm species assignments of collected samples. In addition, we developed and validated a multiplex real-time PCR assay to rapidly differentiate *C. pomonella* and *G. molesta* using species-specific polymorphisms in the mitochondrial COI region. The developed assay provides a practical molecular diagnostic tool for rapid species discrimination and may support phytosanitary surveillance, quarantine inspection, and integrated pest management programs in Kazakhstan and neighboring fruit-growing regions.

## 2 Materials and methods

### 2.1 Sampling of fruit-infesting Tortricids and their morphological identification

Field studies were conducted from September 2024 to April 2026 in seven apple orchards of the southern and southeastern regions of Kazakhstan. Additionally, the mixed orchard was inspected, and several specimens were collected, including *Grapholita funebrana*, which predominantly infests stone fruits (22). *G. funebrana* specimens were included in the study as a closely related non-target species to assess the specificity of both the molecular identification approach and the developed qPCR assay (**Table 1**). To detect all possible apple moth populations in orchards, quarantine phytosanitary surveys were conducted using pheromone traps, trap bands, and visual inspection of fruits. The geographic coordinates of each sampling point were recorded using a Garmin Montana 750i device. GPS-tagged point mapping was performed in QGIS 3.28 using the Lat Lon Tools plugin.

**Table 1:** Surveyed apple orchard populations with coordinates and detected species.

No. on map	Sampling location	Coordinates	Number of specimens	Type
1	Turkestan Region	42.438952003 69.790557410	4	*Сydia pomonella*
2	Almaty Region	43.364106408 77.477838505	14	*Сydia pomonella*
3	Zhetysu Region	45.904680761 80.555252462	6	*Сydia pomonella*
4	Zhetysu Region	45.486416768 80.499568447	3	*Сydia pomonella*
5	Almaty Region	43.522500862 76.833057960	23	*Сydia pomonella*
6	Almaty Region	43.445000851 77.450557953	2 1	*Сydia pomonella* *Grapholita molesta*
7	Almaty Region	43.290158858 77.178464394	3	*Grapholita funebrana*
			7	*Grapholita molesta*
			3	*Сydia pomonella*

Morphological identification of larvae was carried out using a Leica IVESTA3 stereomicroscope. The analysis considered a set of differential characters, including body coloration, larval size, the pattern of setae distribution, and the presence and development of the anal comb (23, 24).

### 2.2 COI-based amplification, sequencing, and molecular species identification

Genomic DNA was extracted using a modified CTAB (cetyltrimethylammonium bromide) method adapted for insects (25). The concentration and purity of the extracted DNA were assessed using a NanoDrop spectrophotometer (Thermo Scientific, USA).

For amplification, primers specific to the COI region were used (5’-TCCACTAATCACAARGATATTGGTAC-3’, 5’-GAAAATCATAATGAAGGCATGAGC-3’) (26). The reaction conditions were as follows: 1 U Taq DNA Polymerase (New England Biolabs), 1X Taq buffer, 0.2 µM dNTPs, 0.2 µM of each primer, and 20 ng of DNA template in a final volume 25µl. Amplification was performed under the following program: initial denaturation at 95 °C for 2 minutes; followed by 5 cycles consisting of denaturation at 95 °C for 1 minute, primer annealing at 46 °C for 1 minute, and extension at 72 °C for 30 seconds. This was followed by 35 cycles of 95 °C for 1 minute, annealing at 53 °C for 1 minute, and extension at 72 °C for 30 seconds. A final extension step was performed at 72 °C for 5 minutes. Amplification products were visualized by electrophoresis in 1.5% agarose gel stained with ethidium bromide. Gel images were captured and analyzed using a ChemiDoc imaging system (Bio-Rad).

The sequencing of COI amplicons was performed on a MinION Mk1B device (Oxford Nanopore Technologies, UK). Quantification of amplicons was performed using a Qubit Flex fluorometer (Thermo Fisher Scientific, USA). Library preparation was carried out using the Rapid Barcoding Kit 96 (SQK-RBK110.96, Oxford Nanopore Technologies, Oxford, UK) according to the manufacturer’s protocol.

Raw sequencing data were basecalled in MinKNOW (v25.05.14) using the integrated Dorado basecaller (v7.9.8), and reads with mean Phred quality scores below 10 were removed using NanoFilt. Quality-filtered reads were initially mapped with minimap2 to a curated reference database comprising approximately 32,000 Tortricidae mitochondrial sequences retrieved from NCBI to identify the most suitable reference sequence for each sample. For each specimen, reads were subsequently remapped to the selected reference using minimap2, and consensus sequences were generated from the resulting alignments using SAMtools. The consensus sequences were then inspected, trimmed, and aligned in UGENE v.50.0 (27) using the MAFFT algorithm. The consensus sequences were 139 bp in length in all analyzed specimens after quality trimming and alignment processing. Final COI consensus sequences were queried against the NCBI nucleotide database using BLASTn to assign species identity based on sequence similarity and alignment coverage.

For phylogenetic analysis, sequences for 15 species of *Tortricidae* infesting apple were retrieved from the NCBI database, **Table 2**. Only sequences overlapping the target region (139 bp) amplified in this study were retained for analysis. Sequences that did not cover the amplified fragment were removed to ensure comparability and accurate downstream analyses.

**Table 2:** COI sequences of *Tortricidae* retrieved from NCBI GenBank.

Species	Number of accessions
*Сydia pomonella*	296
*Grapholita molesta*	89
*Grapholita_funebrana*	41
*Hedya_nubiferana*	40
*Pandemis_cerasana*	39
*Pandemis_heparana*	38
*Archips_argyrospila*	35
*Adoxophyes_orana*	34
*Archips_podana*	26
*Cydia_splendana*	24
*Archips_xylosteana*	22
*Argyrotaenia_ljungiana*	19
*Archips_rosana*	18
*Spilonota_lechriaspis*	17
*Archips_crataegana*	16
*Pandemis_pyrusana*	2

For each species, multiple sequence alignments were performed using MAFFT, and consensus sequences were generated from the resulting alignments. These consensus sequences were then used for downstream phylogenetic analysis. Phylogenetic trees were reconstructed using the maximum likelihood method implemented in IQ-TREE, with model selection performed by ModelFinder. Branch support was assessed using 1000 ultrafast bootstrap and SH-aLRT replicates. Further editing and visualization of the tree were performed in iTOL v.7 (28).

### 2.3 Design of primers and probes and qPCR detection of *Cydia pomonella* and *Grapholita molesta*

Primers and probes were designed using species-specific consensus sequences derived from COI gene alignments for phylogenetic analysis. To ensure high assay specificity and minimize the risk of false-positive amplification, a dataset comprising COI sequences from a broad range of *Tortricidae* representatives infesting apple was assembled and analyzed. Aligned COI sequences were screened to identify conserved regions suitable for primer binding and variable regions containing diagnostic polymorphisms. Particular emphasis was placed on identifying nucleotide positions that distinguish the target species from closely related *Tortricidae* taxa. Primer and probe design were performed using Geneious Prime v. 2025.2.1. Design parameters followed commonly accepted criteria for qPCR assay development, including optimal primer length, GC content, and melting temperature, as well as avoiding secondary structures such as hairpins, self-dimers, and heterodimers (29). Probes were designed to target species-specific polymorphic sites, further increasing assay specificity.

In silico specificity analysis of primers and probes was performed in Geneious Prime v. 2025.2.1. by aligning candidate oligonucleotide sequences against the *Tortricidae* reference dataset generated in this study. Primer- and probe-binding sites were manually examined for diagnostic nucleotide mismatches between target and non-target taxa.

qPCR was performed using the Luna Universal Probe qPCR Master Mix in a final reaction volume of 20 µL. The reaction mixture contained 1× master mix, forward and reverse primers for target organisms, and an internal control at a final concentration of 0.4 µM each, hydrolysis probes at a final concentration of 0.2 µM (**Table 3**), template DNA at different dilutions ranging from 50 ng to 0.1 pg, and nuclease-free water.

**Table 3:** Description of primers and probes for the detection of *Cydia pomonella* and *Grapholita molesta*.

Target species/control	Name	Type	Sequence (5′–3′)	Label	Reference
*Cydia pomonella/Grapholita molesta* (COI gene)	CG_fw	Primer	TTTGGWATTTGAGCYGGWATAGTAGG	—	This study
	CG_rv	Primer	GCAGTWACAATAGTATTATAAATTTGATC	—	
*Cydia pomonella* (COI gene)	Cp_probe	Probe	GTAATCTTAGAGAAGTTCC	FAM–MGB	
*Cydia pomonella/Grapholita molesta* (COI gene)	Cp_Gm probe	Probe	ATTCGAGCAGAATTAGG	Cy5–MGB	
*Grapholita molesta* (COI gene)	Gm_probe	Probe	ACCTGGATTACCTAATTC	HEX–MGB	
Internal control (28S rRNA gene)	UNI28S-fwd	Primer	CTACTATCTAGCGAAACC	—	(30)
	UNI28S-rev	Primer	AYTAGAGTCAAGCTCAAC	—	
	UNI28S-P	Probe	AAA+G+A+AG+A+C+C+C+T	Texas Red–BHQ-2	

W, A or T nucleotide; Y, C or T nucleotide; +, LNA.

Amplification was performed on the QuantStudio 5 Real-Time PCR System using the following cycling conditions: an initial denaturation at 95 °C for 3 min, followed by 40 amplification cycles consisting of denaturation at 95 °C for 15 s and annealing/extension at 56 °C for 30 s. Fluorescence signals were recorded at each cycle in the FAM, HEX, Cy5, and Texas Red channels.

### 2.4 Statistical analysis

Descriptive statistics, including mean Ct values, standard deviations (SD), coefficients of variation (CV, %), and detection rates (%), were calculated for all qPCR assays. Linear regression analysis was performed for standard curve construction and calculation of amplification efficiency and coefficient of determination (R²) according to MIQE recommendations (31).

## 3 Results

### 3.1 Sampling and COI-based molecular identification of Tortricids

**Figure 1:**
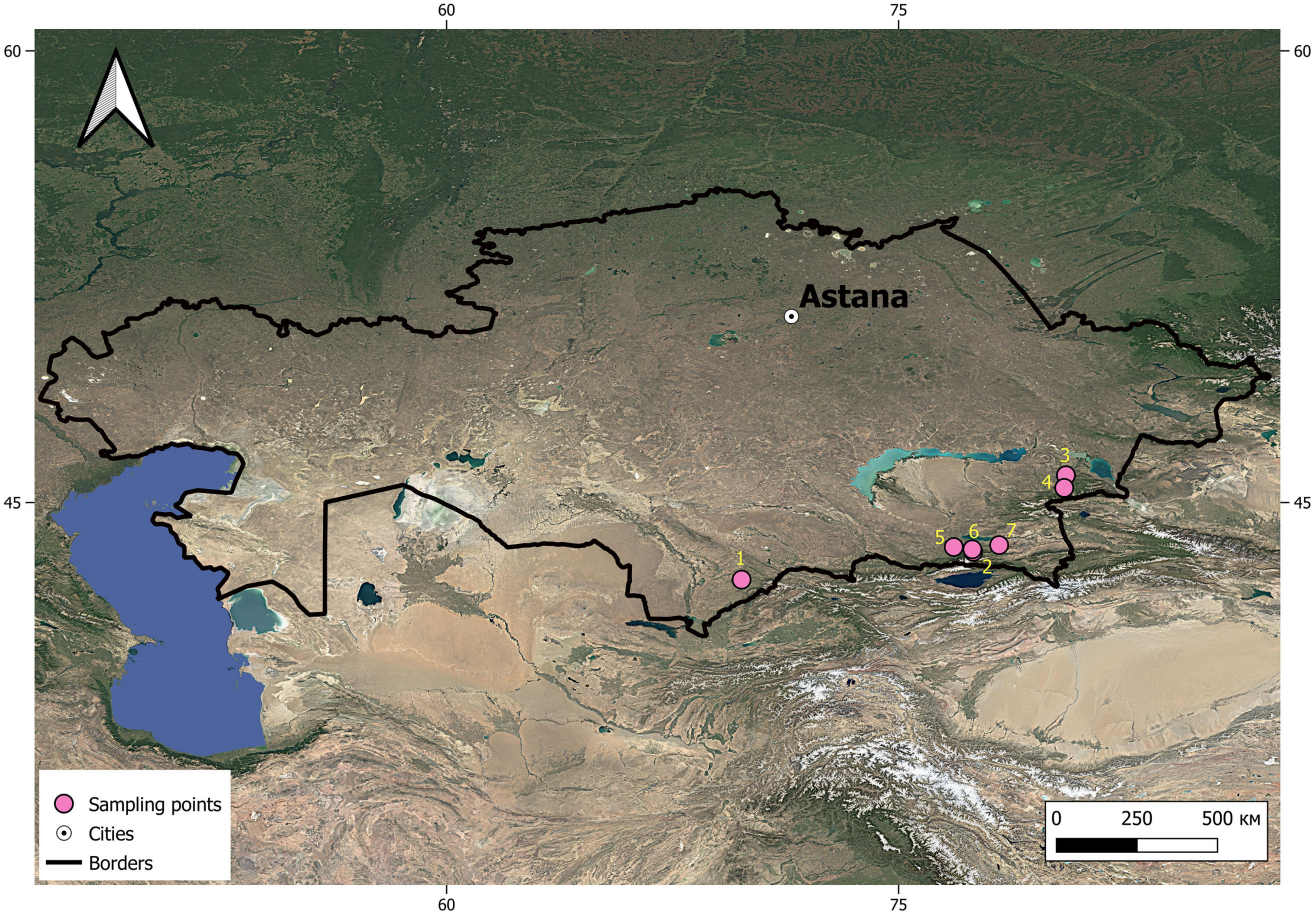
Map of the sampling regions and collection sites used in the present study.

Sampling of Tortricids in apple orchards in southeastern and southern Kazakhstan was conducted during 2024 - 2026. The study covered three large regions of Kazakhstan, which are the main areas of industrial apple production in the country (**Table 1**, **Figure 1**). A total of 66 specimens were collected. The majority of the sample consisted of *C. pomonella* individuals (n = 55), detected in all studied regions. In the Almaty Region, the following species were also identified: *G. funebrana* (n = 3) in mixed orchard and *G. molesta* (n = 8).

**Figure 2:**
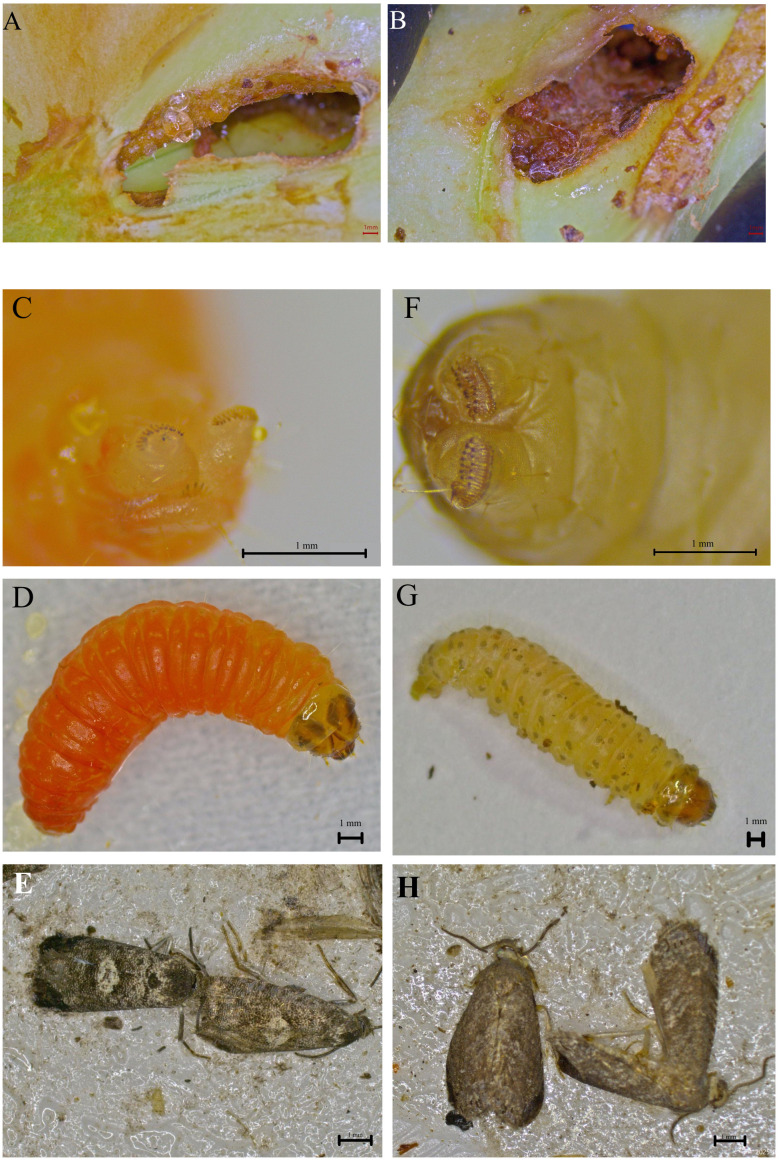
Morphological features and types of fruit damage caused by codling moth larvae. **(A, B)** – typical damage of apple fruits with larval galleries and frass in the seed chamber area; **(C, D, E)** – morphological characteristics of *Grapholita molesta*; **(F, G, H)** – morphological characteristics of *Cydia pomonella*.

Initial species assignment was performed using morphological characters of both larval and adult specimens (**Figure 2**) and was subsequently verified by COI-based molecular identification. During visual examination of infested fruits, typical damage symptoms were observed, including the presence of entry holes filled with frass and plant debris, as well as internal tunnels within the fruit tissues leading to the seed chamber. This type of damage is characteristic of larvae of *C. pomonella* and other representatives of the family *Tortricidae* and served as a primary indicator of pest presence (32).

In larvae, identification was based on body coloration, degree of head capsule sclerotization, structure of the anal plate, terminal abdominal segments, and chaetotaxy. Particular attention was paid to diagnostic characters separating *C. pomonella* and *G. molesta*, including the arrangement of D1 and SD1 setae on the ninth abdominal segment, the position of the L group setae on the first thoracic segment, the fusion of MSD1 and MSD2 pinacula on the mesothorax, and the structure of crochets on abdominal segments 3–6. In G. molesta, the presence of a black anal comb above the anal opening was used as an additional diagnostic character distinguishing it from the morphologically similar *C. pomonella*. Despite the use of these characters, larval identification remained challenging due to variation in body pigmentation, ranging from light cream to pink and orange, and due to the small size or partial damage of some specimens. When compared with COI-based molecular identification, morphological identification of larvae showed 80% concordance, indicating that larval morphology is useful for preliminary screening but may be insufficient for reliable species-level discrimination in all cases.

Adult specimens were identified using external morphological characters, including forewing coloration, wing pattern, and general body morphology. Compared with larvae, adults showed higher diagnostic reliability; however, identification was still affected by specimen conditions, particularly when individuals were collected from traps or had partially damaged wing scales. Comparison with molecular identification showed 89% concordance for adult specimens, confirming that adult morphology is more informative than larval morphology but still benefits from molecular confirmation.

**Figure 3:**
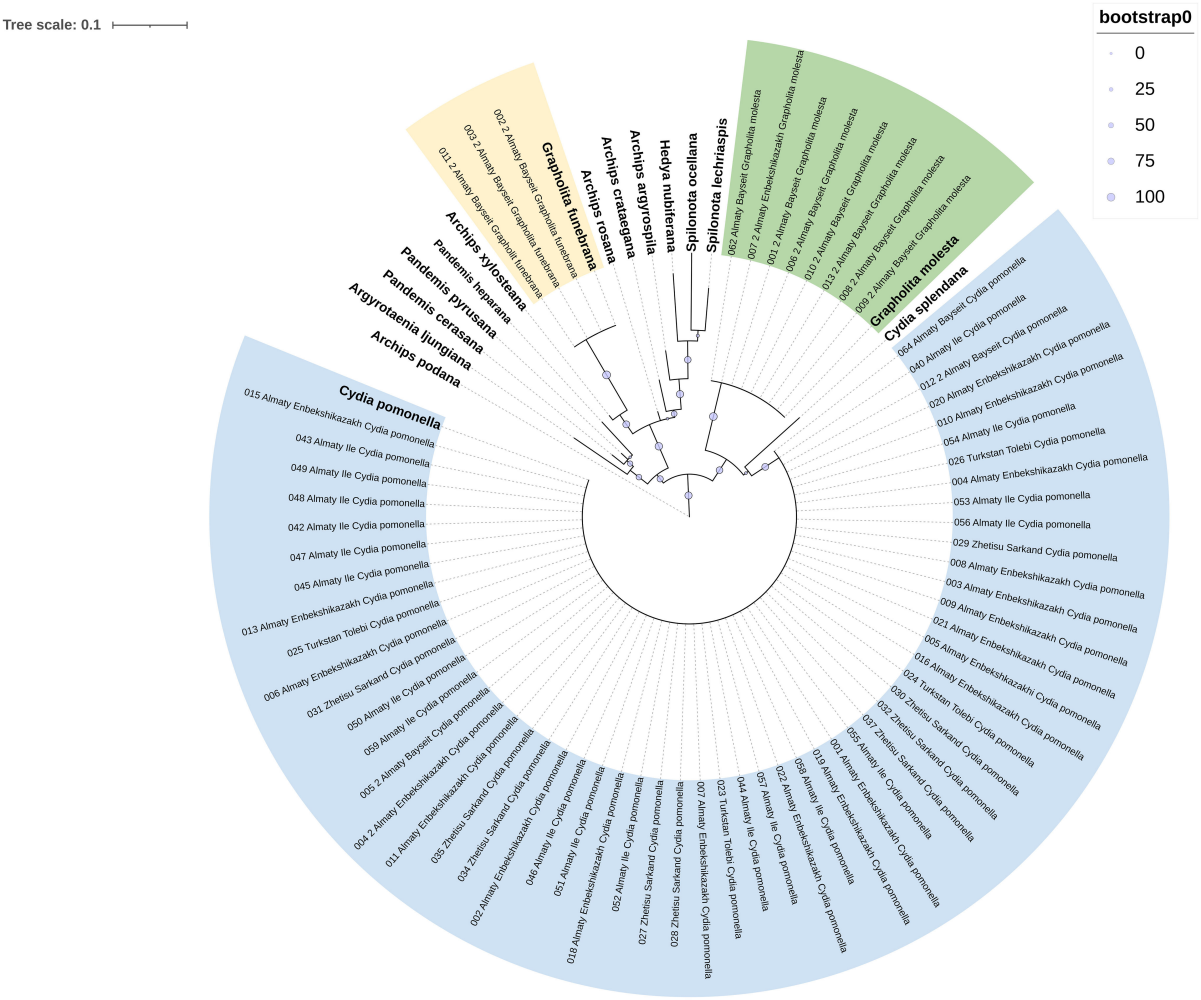
Phylogenetic tree of samples based on a fragment of the COI gene. The blue color indicates the *C. pomonella* group, the green color indicates the *G. molesta* group, and the orange color indicates the *G. funebrana* group; the point gradient reflects the bootstrap support for nodes.

All COI sequences of specimens obtained in this study were uploaded to the NCBI database under the following accession numbers: PZ477474-PZ477523. Molecular analysis of COI sequences enabled reliable differentiation of the collected tortricid specimens and their assignment to reference sequences from GenBank. Following quality filtering, all consensus sequences were trimmed to 139 bp to ensure consistent alignment across analyzed specimens prior to phylogenetic reconstruction. The resulting COI-based clustering analysis separated the analyzed specimens into three species-specific groups corresponding to *C. pomonella*, *G. molesta*, and *G. funebrana* (**Figure 3**). The tree was generated exclusively for species-level confirmation and visualization of taxonomic clustering and was not intended for reconstruction of evolutionary relationships or population structure. Most specimens collected from apple orchards in Kazakhstan clustered with *C. pomonella*, which was detected in all surveyed regions. In contrast, *G. molesta* and *G. funebrana* were detected only in Almaty Region, including *G. funebrana* samples collected from a mixed orchard. These results indicate that *C. pomonella* remains the dominant tortricid species in the surveyed apple orchards, while the detection of *G. molesta* confirms the presence of this quarantine-relevant species in southeastern Kazakhstan.

Although the analyzed COI fragment was relatively short, it contained sufficient interspecific variation to distinguish the 16 species of Tortricids infesting the apple. At the same time, the limited sequence length reduced the resolution of intraspecific relationships, as reflected in weak structuring within subclades. Therefore, the COI fragment used in this study was suitable for species-level confirmation but less informative for detailed population-level analysis.

### 3.2 qPCR identification of *C. pomonella* and *G. molesta*

**Figure 4:**
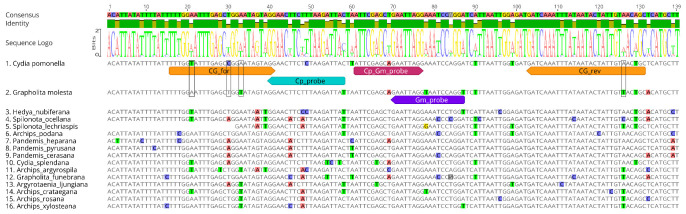
Primers and probes for *C. pomonella* and *G. molesta* detection by qPCR. The orange arrows showed forward and reverse primers for target region amplification. Variable nucleotides in primer sequences are framed in a rectangle. The red arrow Cp_Gm_probe is specific for both *C. pomonella* and *G. molesta* species. Emerald arrow Cp_probe is specific for *C. pomonella*, where the purple arrow is for *G. molesta*.

Primers and probes were designed for the simultaneous detection and discrimination of *C. pomonella and G. molesta* based on a comparative analysis of mitochondrial COI sequences used in phylogenetic analysis in this study. A single pair of degenerate primers was designed to amplify a shared COI fragment in both target species, while three hydrolysis probes were developed: two species-specific probes and one probe targeting a conserved region shared by both species. The probe design strategy enabled both species discrimination and internal confirmation of *Tortricidae* DNA in a single reaction. As shown in the alignment (**Figure 4**), primer binding sites were located within highly conserved regions, whereas probes targeted polymorphic positions enabling reliable differentiation between closely related taxa. The use of MGB-modified probes allowed the design of short, highly specific probes targeting diagnostic nucleotide substitutions. The variable nucleotides in primer regions were replaced with W for A or T and Y for C or T.

The assay interpretation was based on a combined probe-signal pattern. Samples of *C. pomonella* were identified by simultaneous amplification in the FAM and Cy5 channels, whereas *G. molesta* was identified by amplification in the HEX and Cy5 channels. A Cy5-only signal was interpreted as amplification of a closely related non-target *Grapholitini* species rather than as a positive result for either target species. In the specificity panel, this pattern was observed for *G. funebrana*, indicating that the Cy5 probe targets a conserved region shared among closely related taxa, while the FAM and HEX probes provide species-level discrimination.

The assay’s analytical sensitivity was evaluated using serial dilutions of genomic DNA from 50 ng to 0.1 pg per reaction. For *C. pomonella*, consistent amplification (100% detection rate) was observed down to 5 pg, with mean Ct values increasing proportionally as DNA concentration decreased (**Table 4**). At 1 pg, the detection rate decreased to 66%, indicating proximity to the analytical limit of detection (LOD), whereas no amplification was observed at 0.1 pg.

**Table 4:** Sensitivity of the multiplex qPCR assay using genomic DNA dilutions.

Species	DNA amount per reaction	FAM Ct, mean ± SD	HEX Ct, mean ± SD	Cy5 Ct, mean ± SD	Positive replicates	Detection rate (%)	CV (%)	Interpretation
*C. pomonella*	5 ng	22.54 ± 0.21	—	23.57 ± 0.22	3/3	100	0.93	Positive
	500 pg	26.12 ± 0.27	—	25.88 ± 0.26	3/3	100	1.03	Positive
	50 pg	29.61 ± 0.36	—	29.34 ± 0.33	3/3	100	1.22	Positive
	5 pg	32.39 ± 0.49	—	35.11 ± 0.47	3/3	100	1.51	Positive
	1 pg	39.52 ± 1.21	—	36.92	2/3	66	3.06	Near LOD
	0.1 pg	—	—	—	0/3	0	—	Not available
*G. molesta*	5 ng	—	23.62 ± 0.24	23.57 ± 0.22	3/3	100	1.02	Positive
	500 pg	—	26.65 ± 0.29	25.88 ± 0.26	3/3	100	1.09	Positive
	50 pg	—	29.89 ± 0.39	29.34 ± 0.33	3/3	100	1.30	Positive
	5 pg	—	34.06 ± 0.53	35.11 ± 0.47	3/3	100	1.56	Positive
	1 pg	—	37.18	36.92	1/3	33	n.c	Near LOD
	0.1 pg	—	—	—	0/3	0	—	Not available

CV, coefficient of variation; SD, standard deviation; LOD, analytical limit of detection; nc, not calculated.

A similar pattern was observed for *G. molesta*, with reliable detection down to 5 pg (100%), whereas at 1 pg reproducibility decreased to 33%. No amplification was observed at a concentration of 0.1 pg.

At DNA concentrations ranging from 50 ng to 5 pg per reaction, all technical replicates produced amplification signals, resulting in a 100% detection rate for both target species. Ct variation between replicates remained low throughout this range, with coefficients of variation (CV) generally below 1.56%, indicating high repeatability of the assay. For *C. pomonella*, CV values ranged from 0.93% to 1.51% at concentrations between 50 ng and 5 pg, with increased variability near the limit of detection, reaching 3.06% at 1 pg. Similarly, for *G. molesta*, CV values ranged from 1.02% to 1.56% within the same concentration range. For the 0.1 pg concentration, CV was not calculated due to the absence of amplification.

**Figure 5:**
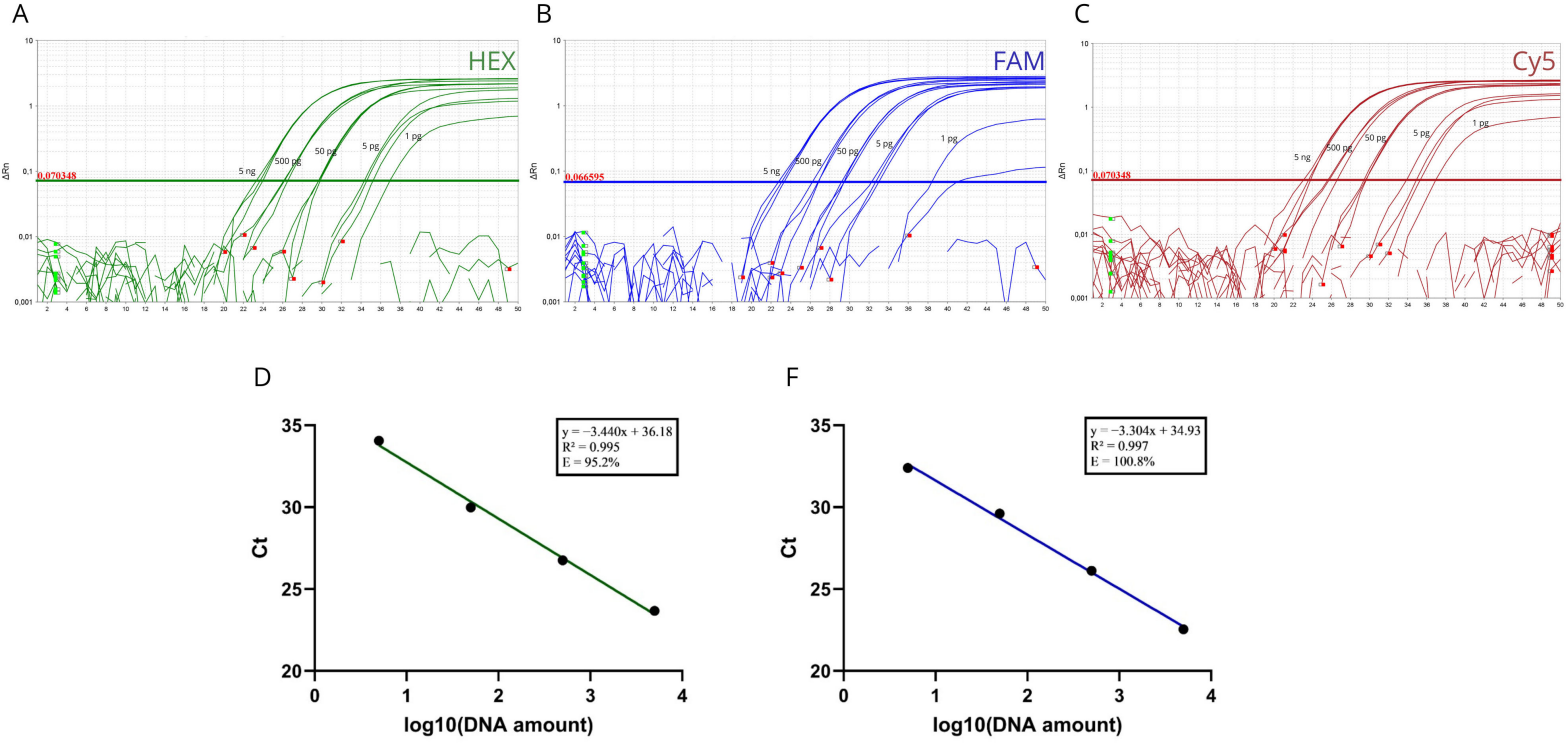
Real-time PCR amplification and standard curve analysis for target pest species. **(A–C)** – Serial dilutions of genomic DNA were analysed in three technical replicates; **(D, C)** – Standard curves constructed from log10-transformed DNA concentrations demonstrate linear relationships between Ct values and template input for both target species.

Standard curve analysis within the assay’s linear dynamic range (50 ng–5 pg) demonstrated high amplification efficiency and assay linearity for both target species. For *C. pomonella*, the standard curve showed a slope of −3.304, an amplification efficiency of 100.8%, and a coefficient of determination (R²) of 0.997, **Figure 5**. Similarly, the *G. molesta* assay demonstrated a slope of −3.440, amplification efficiency of 95.2%, and an R² value of 0.995. Ct values increased proportionally with decreasing DNA concentration, confirming stable amplification kinetics and reliable quantitative performance across the tested dilution series. Lower DNA concentrations (1 pg and 0.1 pg) were excluded from regression analysis due to stochastic amplification effects near the detection limit. Overall, the assay demonstrated high sensitivity, with a analytical detection limit of approximately 5–50 pg of DNA per reaction, depending on the target species.

Specificity was evaluated against a panel of closely related and non-target species, including *G. funebrana*, *Cydia splendana*, *Archips podana*, *Halyomorpha halys*, *Lymantria dispar*, and *Quadraspidiotus perniciosus*, **Table 5**. The assay showed no false-positive amplification in any non-target samples at 5 or 50 pg.

**Table 5:** Specificity and cross-reactivity evaluation of the multiplex qPCR assay.

Species	n	Expected result	FAM+Cy5 (Cp, n)	HEX+Cy5 (Gm, n)	FAM (n)	HEX (n)	Cy5 (n)	False positives	Detection rate (%)
*Cydia pomonella*	10	FAM + Cy5	10	0	10	0	0	0	100
*Grapholita molesta*	8	HEX + Cy5	0	8	0	8	0	0	100
*Grapholita funebrana*	3	Cy5 only	0	0	0	0	3	0	0
*Cydia splendana*	6	Negative	0	0	0	0	0	0	0
*Archips podana*	9	Negative	0	0	0	0	0	0	0
*Halyomorpha halys*	8	Negative	0	0	0	0	0	0	0
*Lymantria dispar*	7	Negative	0	0	0	0	0	0	0
*Quadraspidiotus perniciosus*	6	Negative	0	0	0	0	0	0	0

Cp, Cydia pomonella; Gm, Grapholita molesta.

## 4 Discussion

The present study provides the first integrated molecular survey of fruit-infesting tortricids in apple orchards of southern and southeastern Kazakhstan, combining field monitoring, morphological assessment, COI sequencing, and multiplex qPCR-based species discrimination. The results refine the current understanding of tortricid species composition associated with apple production in Kazakhstan and provide a practical molecular framework for phytosanitary monitoring. The predominance of *C. pomonella* observed in this study is consistent with its established role as the principal tortricid pest of apple orchards worldwide. Among the 66 specimens collected, *C. pomonella* accounted for 83.3% of the total sample and was detected across all surveyed regions, confirming its broad geographic distribution in Kazakhstan. This pattern aligns with previous studies that describe *C. pomonella* as the dominant internal fruit-feeding pest in pome fruit orchards across Eurasia, with stable populations maintained by high ecological adaptability and increasing resistance to conventional chemical control (10, 33, 34). The results should be interpreted within the scope of the present sampling design, as the number of specimens analyzed was limited and the study focused on the molecular identification of collected individuals rather than a comprehensive assessment of pest population dynamics or regional abundance. The results should also be interpreted in the context of intensive pest management practices widely implemented in commercial apple orchards. As in many fruit-producing regions worldwide, insecticides are routinely used in Kazakhstan to suppress tortricid populations. Such management practices can substantially influence local pest abundance and detection rates during field surveys, potentially obscuring the true distribution and population dynamics of target species. Consequently, the observed occurrence of tortricid species reflects the conditions present at the time of sampling and should not be considered a direct estimate of long-term regional prevalence. More extensive surveys across multiple years, orchards, and management regimes will be required to obtain a comprehensive picture of tortricid distribution and population trends in Kazakhstan. Furthermore, intensive insecticide-based management of codling moth and other tortricid pests is common not only in Kazakhstan but also in most commercial apple-growing regions worldwide (10). As a result, field collections often represent populations that have already been subjected to varying levels of control pressure, which may influence species abundance, detection probability, and apparent distribution patterns.

The detection of *G. molesta* exclusively in the Almaty Region is particularly important from a quarantine perspective. Although represented by fewer specimens than *C. pomonella*, the confirmed occurrence of *G. molesta* supports previous phytosanitary records indicating restricted but persistent establishment of this species in southeastern Kazakhstan (*Grapholita molesta* (LASPMO)[Overview]| EPPO Global Database, n.d.). Because *G. molesta* is regulated as a quarantine pest in Kazakhstan, reliable species confirmation is critical for both national surveillance and movement of planting material and fruit products.

The co-occurrence of C. pomonella and G. molesta has been reported in several apple-growing regions worldwide and represents an important challenge for monitoring and integrated pest management (35, 36). Our findings confirm that both species also occur in southeastern Kazakhstan, highlighting the need for simultaneous molecular detection in regional phytosanitary surveillance. A particularly important finding of this study is the measured concordance between morphological and molecular identification. Morphological identification showed 80% agreement with COI confirmation in larvae and 89% agreement in adults. These results demonstrate that morphology remains a highly informative and practical tool for routine preliminary screening, especially in adult specimens. At the same time, the reduced agreement observed for larvae confirms that species-level diagnosis based solely on larval morphology may remain uncertain in part of the material, particularly when specimens are immature, damaged, or collected from infested fruit tissue. Under phytosanitary inspection conditions, molecular confirmation therefore represents an important complementary diagnostic step rather than a replacement for morphology when intact specimens are available.

The developed assay may also have potential applications for the analysis of mixed biological material or damaged specimens where morphological identification is difficult. Similar approaches have been proposed for arthropod monitoring using environmental DNA (37–39), although this application was not evaluated in the present study.The COI mini-barcode used in this study proved sufficient for reliable species-level discrimination among the analyzed tortricids. Although the final alignment length was limited to 139 bp after quality trimming, the amplified region contained sufficient diagnostic nucleotide variation to distinguish Cydia pomonella, Grapholita molesta, and Grapholita funebrana. These findings demonstrate that even a relatively short COI fragment can provide reliable molecular confirmation of morphologically similar tortricid species when the objective is species identification rather than evolutionary inference. The obtained results are consistent with previous studies demonstrating the usefulness of COI barcoding for the identification of economically important tortricid pests, particularly when larval morphology is ambiguous or specimens are damaged. COI-based molecular identification has been successfully applied in quarantine diagnostics and fruit export inspections to distinguish closely related fruit moth species (18). Likewise, COI sequencing has been shown to facilitate the identification of cryptic Grapholita species that cannot be reliably separated using morphology alone (40). At the same time, the short COI fragment used in the present study has limited value for population genetic or evolutionary analyses. Although it was sufficient for species confirmation, its phylogenetic resolution within species was low. Previous studies have demonstrated that analyses of population structure, invasion history, and gene flow in Grapholita molesta and Cydia pomonella require more informative nuclear markers, such as microsatellites or genome-wide SNPs (41). Therefore, the COI fragment used in the present study should be interpreted primarily as a diagnostic marker for species identification, whereas future studies investigating population diversity and dispersal of tortricid pests in Kazakhstan should employ higher-resolution nuclear markers or genome-wide approaches.

The multiplex qPCR assay developed in this study represents the main applied outcome of the work. In contrast to previously reported diagnostic systems based on conventional PCR, PCR-RFLP, or sequencing confirmation, the developed assay enables simultaneous detection and differentiation of *C. pomonella* and *G. molesta* within a single reaction using a shared degenerate primer pair and three hydrolysis probes. The previously developed qPCR-based test system is available only for the rapid identification of *C. pomonella* and was used on intercepted material at U.S. ports of entry, noting that immature larvae are often difficult to identify morphologically and that molecular confirmation can reduce unnecessary quarantine actions (21). The developed test system in the present study provides both species-specific discrimination and internal confirmation of *Grapholitini* DNA. The Cy5-only signal observed for *G. funebrana* is particularly informative diagnostically, as it allows recognition of closely related non-target *Grapholitini* without false assignment to either target species. Analytical validation demonstrated high assay sensitivity and reproducibility. Both target species showed consistent amplification in all replicates down to 5 pg of genomic DNA per reaction, with a 100% detection rate across this range. At 1 pg, amplification became less reproducible, indicating that amplification was approaching the limit of detection. Therefore, the analytical detection limit of the assay can be considered to be approximately 5 pg of DNA per reaction under the tested conditions. Standard curve parameters further confirmed robust assay performance, with amplification efficiencies of 95.2–100.8% and strong linearity (R² = 0.995–0.997), thereby fully supporting the assay’s quantitative reliability. Specificity testing against closely related Tortricidae and unrelated non-target insects further confirmed assay robustness. No false-positive amplification was detected among C*. splendana, A. podana, H. halys, L. dispar, or Q. perniciosus*. High analytical specificity is especially important in orchard monitoring systems where multiple pest species may occur simultaneously, and mixed biological material is frequently encountered. The present study demonstrates that multiplex COI-based qPCR provides a reliable, sensitive, and operationally practical approach for rapid differentiation of economically important fruit-infesting tortricids. The developed assay could be a useful diagnostic tool for phytosanitary surveillance, quarantine inspections, and integrated pest management programs, especially in orchards, where *C. pomonella* and *G. molesta* coexist.

From a practical perspective, the results support implementing an integrated diagnostic workflow for codling moth monitoring in Kazakhstan, based on three complementary stages: pheromone-based surveillance for early pest detection, morphological sorting for rapid preliminary screening, and molecular confirmation via multiplex qPCR for quarantine-relevant or diagnostically ambiguous samples. Such an approach may substantially improve the precision of phytosanitary diagnostics, reduce uncertainty during species identification, and support more timely pest management decisions in commercial orchards.

## 5 Conclusions

The present study clarifies the species composition of the codling moth complex in apple orchards of southern and southeastern Kazakhstan and confirms the dominant occurrence of *C. pomonella*, while also documenting the presence of the quarantine-relevant species *G. molesta*. COI-based molecular identification enabled reliable species discrimination and confirmed its value as a rapid diagnostic marker for *Grapholitini*. Based on diagnostic polymorphisms in the COI region, a multiplex qPCR assay was developed to simultaneously detect and differentiate *C. pomonella* and *G. molesta*, demonstrating high specificity, sensitivity, and reproducibility.

Overall, the results highlight the practical value of molecular diagnostics for phytosanitary monitoring, early detection, and reliable identification of fruit-infesting tortricid pests in apple orchards.

## Data Availability

The datasets presented in this study can be found in online repositories. The names of the repository/repositories and accession number(s) can be found below: https://www.ncbi.nlm.nih.gov/genbank/, PZ477474-PZ477523.

## Supplementary material

File S1 – List of accession numbers of sequences from the NCBI database used to construct the phylogenetic tree.
