# Supplementary material for: Molecular monitoring and multiplex qPCR-based detection of Cydia pomonella and Grapholita molesta in apple orchards of Kazakhstan
Source: Front Insect Sci. 2026 Jul 31;6:1897268. doi: 10.3389/finsc.2026.1897268 (PMC13473341; doi:10.3389/finsc.2026.1897268)
Supplement: Supplementary file 1 [file DataSheet1.docx]

**List of accession numbers of sequences from the NCBI database used to construct the phylogenetic tree**

*Adoxophyes orana*

HM876048.1 Adoxophyes orana voucher MM14376 cytochrome oxidase subunit 1 (COI) gene, partial cds; mitochondrial

HM902023.1 Lepidoptera sp. BOLD:AAD8062 voucher BC ZSM Lep 23426 cytochrome oxidase subunit 1 (COI) gene, partial cds; mitochondrial

HM871363.1 Adoxophyes orana voucher MM02007 cytochrome oxidase subunit 1 (COI) gene, partial cds; mitochondrial

HM871769.1 Adoxophyes orana voucher MM02980 cytochrome oxidase subunit 1 (COI) gene, partial cds; mitochondrial

HQ955195.1 Adoxophyes orana voucher BC ZSM Lep 25305 cytochrome oxidase subunit 1 (COI) gene, partial cds; mitochondrial

JF733846.1 Adoxophyes orana voucher O-1 cytochrome oxidase subunit I (COI) gene, partial cds; mitochondrial

JF733847.1 Adoxophyes orana voucher O-2 cytochrome oxidase subunit I (COI) gene, partial cds; mitochondrial

JF733848.1 Adoxophyes orana voucher O-143 cytochrome oxidase subunit I (COI) gene, partial cds; mitochondrial

JF733849.1 Adoxophyes orana voucher O-144 cytochrome oxidase subunit I (COI) gene, partial cds; mitochondrial

JF733850.1 Adoxophyes orana voucher O-145 cytochrome oxidase subunit I (COI) gene, partial cds; mitochondrial

JF733851.1 Adoxophyes orana voucher O-N cytochrome oxidase subunit I (COI) gene, partial cds; mitochondrial

JF703015.1 Adoxophyes orana voucher JDDNA4599 cytochrome c oxidase subunit I gene, partial cds; mitochondrial

KF523731.1 Adoxophyes orana voucher KTP-06-0119-2 cytochrome oxidase subunit 1 (COI) gene, partial cds; mitochondrial

KF523732.1 Adoxophyes orana voucher KTP-06-0126-1 cytochrome oxidase subunit 1 (COI) gene, partial cds; mitochondrial

KF523733.1 Adoxophyes orana voucher KTP-06-0119-1 cytochrome oxidase subunit 1 (COI) gene, partial cds; mitochondrial

KF523734.1 Adoxophyes orana voucher KTP-06-0126-2 cytochrome oxidase subunit 1 (COI) gene, partial cds; mitochondrial

KM572053.1 Adoxophyes orana voucher BIOUG04118-B10 cytochrome oxidase subunit 1 (COI) gene, partial cds; mitochondrial

KM572230.1 Adoxophyes orana voucher BIOUG04118-E08 cytochrome oxidase subunit 1 (COI) gene, partial cds; mitochondrial

KM572335.1 Adoxophyes orana voucher BIOUG04118-F07 cytochrome oxidase subunit 1 (COI) gene, partial cds; mitochondrial

KM572497.1 Adoxophyes orana voucher BIOUG04118-C07 cytochrome oxidase subunit 1 (COI) gene, partial cds; mitochondrial

KM572896.1 Adoxophyes orana voucher BIOUG04118-F06 cytochrome oxidase subunit 1 (COI) gene, partial cds; mitochondrial

KM573184.1 Adoxophyes orana voucher TLMF Lep 08012 cytochrome oxidase subunit 1 (COI) gene, partial cds; mitochondrial

KM573373.1 Adoxophyes orana voucher BIOUG04118-B11 cytochrome oxidase subunit 1 (COI) gene, partial cds; mitochondrial

KM573388.1 Adoxophyes orana voucher BIOUG04118-E07 cytochrome oxidase subunit 1 (COI) gene, partial cds; mitochondrial

KX046687.1 Adoxophyes orana voucher BC ZSM Lep 61530 cytochrome oxidase subunit 1 (COI) gene, partial cds; mitochondrial

KX048520.1 Adoxophyes orana voucher NHMO-06132 cytochrome oxidase subunit 1 (COI) gene, partial cds; mitochondrial

KX440768.1 Adoxophyes orana isolate SQ0925069 cytochrome c oxidase subunit I (COI) gene, partial cds; mitochondrial

MF049942.1 Adoxophyes orana isolate DLS110710.150 cytochrome oxidase subunit I (COI) gene, partial cds; mitochondrial

MF050555.1 Adoxophyes orana isolate JG100812.211 cytochrome oxidase subunit I (COI) gene, partial cds; mitochondrial

MN565324.1 Adoxophyes orana isolate 20160101 cytochrome oxidase subunit I (COI) gene, partial cds; mitochondrial

MN565325.1 Adoxophyes orana isolate 20160102 cytochrome oxidase subunit I (COI) gene, partial cds; mitochondrial

MZ607069.1 Adoxophyes orana voucher MM23930 cytochrome oxidase subunit 1 (COI) gene, partial cds; mitochondrial

LC672429.1 Adoxophyes orana COI8-2-2 mitochondrial COX1 gene for cytochrome c oxidase subunit 1, partial cds

LC760211.1 Adoxophyes orana HkMic2013-15 mitochondrial COX1 gene for cytochrome c oxidase subunit 1, partial cds

*Archips argyrospila*

AF308931.1 Archips argyrospila cytochrome oxidase subunit 1 (COI) gene, partial cds; and cytochrome oxydase II (COII) gene, complete cds; mitochondrial genes for mitochondrial products

FJ412155.1 Archips argyrospila voucher UBC-2007-0640 cytochrome oxidase subunit 1 (COI) gene, partial cds; mitochondrial

GU800384.1 Archips argyrospila voucher BIOUG<CAN_:MDOK-1456 cytochrome oxidase subunit 1 (COI) gene, partial cds; mitochondrial

HM415704.1 Archips argyrospila voucher BIOUG<CAN_:09BBELE-2102 cytochrome oxidase subunit 1 (COI) gene, partial cds; mitochondrial

HM426914.1 Archips argyrospila voucher BIOUG<CAN_:09BBLEP-01467 cytochrome oxidase subunit 1 (COI) gene, partial cds; mitochondrial

HM426980.1 Archips argyrospila voucher BIOUG<CAN_:09BBLEP-01535 cytochrome oxidase subunit 1 (COI) gene, partial cds; mitochondrial

HM427010.1 Archips argyrospila voucher BIOUG<CAN_:09BBLEP-01567 cytochrome oxidase subunit 1 (COI) gene, partial cds; mitochondrial

HM427043.1 Archips argyrospila voucher BIOUG<CAN_:09BBLEP-01601 cytochrome oxidase subunit 1 (COI) gene, partial cds; mitochondrial

HM427516.1 Archips argyrospila voucher BIOUG<CAN_:09BBLEP-02082 cytochrome oxidase subunit 1 (COI) gene, partial cds; mitochondrial

HM427527.1 Archips argyrospila voucher BIOUG<CAN_:09BBLEP-02093 cytochrome oxidase subunit 1 (COI) gene, partial cds; mitochondrial

HM427763.1 Archips argyrospila voucher BIOUG<CAN_:09BBLEP-02332 cytochrome oxidase subunit 1 (COI) gene, partial cds; mitochondrial

HM427815.1 Archips argyrospila voucher BIOUG<CAN_:09BBLEP-02385 cytochrome oxidase subunit 1 (COI) gene, partial cds; mitochondrial

HM427821.1 Archips argyrospila voucher BIOUG<CAN_:09BBLEP-02391 cytochrome oxidase subunit 1 (COI) gene, partial cds; mitochondrial

HM427826.1 Archips argyrospila voucher BIOUG<CAN_:09BBLEP-02396 cytochrome oxidase subunit 1 (COI) gene, partial cds; mitochondrial

HM427828.1 Archips argyrospila voucher BIOUG<CAN_:09BBLEP-02398 cytochrome oxidase subunit 1 (COI) gene, partial cds; mitochondrial

HM427867.1 Archips argyrospila voucher BIOUG<CAN_:09BBLEP-02437 cytochrome oxidase subunit 1 (COI) gene, partial cds; mitochondrial

HM428015.1 Archips argyrospila voucher BIOUG<CAN_:09BBLEP-02588 cytochrome oxidase subunit 1 (COI) gene, partial cds; mitochondrial

HQ552838.1 Archips argyrospila voucher BIOUG<CAN_:09BBLEP-00586 cytochrome oxidase subunit 1 (COI) gene, partial cds; mitochondrial

HQ552911.1 Archips argyrospila voucher BIOUG<CAN_:09BBLEP-00661 cytochrome oxidase subunit 1 (COI) gene, partial cds; mitochondrial

HQ985483.1 Archips argyrospila voucher BIOUG<CAN_:10BBLEP-01112 cytochrome oxidase subunit 1 (COI) gene, partial cds; mitochondrial

KM545108.1 Archips argyrospila voucher 08BBLEP-04911 cytochrome oxidase subunit 1 (COI) gene, partial cds; mitochondrial

KM551720.1 Archips argyrospila voucher 08BBLEP-00733 cytochrome oxidase subunit 1 (COI) gene, partial cds; mitochondrial

KT124880.1 Archips argyrospila voucher HLC-23612 cytochrome oxidase subunit 1 (COI) gene, partial cds; mitochondrial

KT125956.1 Archips argyrospila voucher HLC-23692 cytochrome oxidase subunit 1 (COI) gene, partial cds; mitochondrial

KT129085.1 Archips argyrospila voucher 10BBCLP-2745 cytochrome oxidase subunit 1 (COI) gene, partial cds; mitochondrial

KT131051.1 Archips argyrospila voucher 10BBCLP-2786 cytochrome oxidase subunit 1 (COI) gene, partial cds; mitochondrial

KT134093.1 Archips argyrospila voucher 10BBCLP-2746 cytochrome oxidase subunit 1 (COI) gene, partial cds; mitochondrial

KT134261.1 Archips argyrospila voucher 10BBCLP-2785 cytochrome oxidase subunit 1 (COI) gene, partial cds; mitochondrial

KT135165.1 Archips argyrospila voucher HLC-23765 cytochrome oxidase subunit 1 (COI) gene, partial cds; mitochondrial

KT135760.1 Archips argyrospila voucher HLC-23766 cytochrome oxidase subunit 1 (COI) gene, partial cds; mitochondrial

KT136878.1 Archips argyrospila voucher HLC-23764 cytochrome oxidase subunit 1 (COI) gene, partial cds; mitochondrial

KT140789.1 Archips argyrospila voucher 10BBCLP-2789 cytochrome oxidase subunit 1 (COI) gene, partial cds; mitochondrial

MG463736.1 Archips argyrospila voucher BIOUG19867-D04 cytochrome oxidase subunit 1 (COI) gene, partial cds; mitochondrial

MG465717.1 Archips argyrospila voucher BIOUG19867-D02 cytochrome oxidase subunit 1 (COI) gene, partial cds; mitochondrial

PX978566.1 Archips argyrospila isolate 500166.087 cytochrome c oxidase subunit I (COX1) gene, partial cds; mitochondrial

*Archips crataeganus*

GU706517.1 Archips crataeganus voucher BC ZSM Lep 23124 cytochrome oxidase subunit 1 (COI) gene, partial cds; mitochondrial

HM902021.1 Lepidoptera sp. FBLMT170-09 voucher BC ZSM Lep 23400 cytochrome oxidase subunit 1 (COI) gene, partial cds; mitochondrial

KT782384.1 Archips crataeganus voucher MM19904 cytochrome oxidase subunit 1 (COI) gene, partial cds; mitochondrial

KT782488.1 Archips crataeganus voucher MM19262 cytochrome oxidase subunit 1 (COI) gene, partial cds; mitochondrial

KX048201.1 Archips crataeganus voucher MM19263 cytochrome oxidase subunit 1 (COI) gene, partial cds; mitochondrial

KX049165.1 Archips crataeganus voucher MM23223 cytochrome oxidase subunit 1 (COI) gene, partial cds; mitochondrial

KX049183.1 Archips crataeganus voucher NHMO-07031 cytochrome oxidase subunit 1 (COI) gene, partial cds; mitochondrial

MF049746.1 Archips crataeganus isolate DLS110709.122 cytochrome oxidase subunit I (COI) gene, partial cds; mitochondrial

MF049752.1 Archips crataeganus isolate DLS110709.129 cytochrome oxidase subunit I (COI) gene, partial cds; mitochondrial

OQ562580.1 Archips crataeganus voucher INV01079 cytochrome oxidase subunit 1 (COI) gene, partial cds; mitochondrial

PP991410.1 Archips crataeganus isolate ac14 cytochrome c oxidase subunit I (COX1) gene, partial cds; mitochondrial

PV198740.1 Archips crataeganus voucher 2889 cytochrome c oxidase subunit I (COX1) gene, partial cds; mitochondrial

PV198821.1 Archips crataeganus voucher 2989 cytochrome c oxidase subunit I (COX1) gene, partial cds; mitochondrial

PV198824.1 Archips crataeganus voucher 2993 cytochrome c oxidase subunit I (COX1) gene, partial cds; mitochondrial

PV198840.1 Archips crataeganus voucher 3016 cytochrome c oxidase subunit I (COX1) gene, partial cds; mitochondrial

PV203872.1 Archips crataeganus voucher 2060 cytochrome c oxidase subunit I (COX1) gene, partial cds; mitochondrial

*Argyrotaenia ljungiana*

GU706908.1 Argyrotaenia ljungiana voucher BC ZSM Lep 23387 cytochrome oxidase subunit 1 (COI) gene, partial cds; mitochondrial

GU706910.1 Argyrotaenia ljungiana voucher BC ZSM Lep 23404 cytochrome oxidase subunit 1 (COI) gene, partial cds; mitochondrial

GU706911.1 Argyrotaenia ljungiana voucher BC ZSM Lep 23403 cytochrome oxidase subunit 1 (COI) gene, partial cds; mitochondrial

HM872841.1 Argyrotaenia ljungiana voucher MM05462 cytochrome oxidase subunit 1 (COI) gene, partial cds; mitochondrial

HM873173.1 Argyrotaenia ljungiana voucher MM06287 cytochrome oxidase subunit 1 (COI) gene, partial cds; mitochondrial

HM873247.1 Argyrotaenia ljungiana voucher MM06423 cytochrome oxidase subunit 1 (COI) gene, partial cds; mitochondrial

HM902020.1 Lepidoptera sp. BOLD:AAA2955 voucher BC ZSM Lep 23384 cytochrome oxidase subunit 1 (COI) gene, partial cds; mitochondrial

JF859926.1 Argyrotaenia ljungiana voucher TLMF Lep 02375 cytochrome oxidase subunit 1 (COI) gene, partial cds; mitochondrial

JN286471.1 Argyrotaenia ljungiana voucher TLMF Lep 04304 cytochrome oxidase subunit 1 (COI) gene, partial cds; mitochondrial

KM573457.1 Argyrotaenia ljungiana voucher MM21107 cytochrome oxidase subunit 1 (COI) gene, partial cds; mitochondrial

KX043392.1 Argyrotaenia ljungiana voucher UKLB40C01 cytochrome oxidase subunit 1 (COI) gene, partial cds; mitochondrial

KX048318.1 Argyrotaenia ljungiana voucher MM21226 cytochrome oxidase subunit 1 (COI) gene, partial cds; mitochondrial

KX049135.1 Argyrotaenia ljungiana voucher MM21228 cytochrome oxidase subunit 1 (COI) gene, partial cds; mitochondrial

KX049468.1 Argyrotaenia ljungiana voucher MM21227 cytochrome oxidase subunit 1 (COI) gene, partial cds; mitochondrial

KX049608.1 Argyrotaenia ljungiana voucher MM21229 cytochrome oxidase subunit 1 (COI) gene, partial cds; mitochondrial

OQ182949.1 Argyrotaenia ljungiana voucher TLMF Lep 27616 cytochrome oxidase subunit 1 (COI) gene, partial cds; mitochondrial

OQ183152.1 Argyrotaenia ljungiana voucher KLM Lep 14920 cytochrome oxidase subunit 1 (COI) gene, partial cds; mitochondrial

OQ183185.1 Argyrotaenia ljungiana voucher TLMF Lep 29443 cytochrome oxidase subunit 1 (COI) gene, partial cds; mitochondrial

PP991399.1 Argyrotaenia ljungiana isolate al12 cytochrome c oxidase subunit I (COX1) gene, partial cds; mitochondrial

*Cydia pomonella*

GU093184.1 Cydia pomonella voucher BIOUG<CAN_:04HBL006368 cytochrome oxidase subunit 1 (COI) gene, partial cds; mitochondrial

GU093185.1 Cydia pomonella voucher BIOUG<CAN_:04HBL006369 cytochrome oxidase subunit 1 (COI) gene, partial cds; mitochondrial

GU093186.1 Cydia pomonella voucher BIOUG<CAN_:04HBL006556 cytochrome oxidase subunit 1 (COI) gene, partial cds; mitochondrial

GU093187.1 Cydia pomonella voucher BIOUG<CAN_:04HBL006698 cytochrome oxidase subunit 1 (COI) gene, partial cds; mitochondrial

GU095781.1 Cydia pomonella voucher jflandry0412 cytochrome oxidase subunit 1 (COI) gene, partial cds; mitochondrial

GU095782.1 Cydia pomonella voucher jflandry0415 cytochrome oxidase subunit 1 (COI) gene, partial cds; mitochondrial

GU091041.1 Cydia pomonella voucher BIOUG<CAN_:2005-ONT-982 cytochrome oxidase subunit 1 (COI) gene, partial cds; mitochondrial

GU091042.1 Cydia pomonella voucher BIOUG<CAN_:2005-ONT-1326 cytochrome oxidase subunit 1 (COI) gene, partial cds; mitochondrial

GU091043.1 Cydia pomonella voucher BIOUG<CAN_:2005-ONT-1050 cytochrome oxidase subunit 1 (COI) gene, partial cds; mitochondrial

GU091044.1 Cydia pomonella voucher BIOUG<CAN_:2005-ONT-943 cytochrome oxidase subunit 1 (COI) gene, partial cds; mitochondrial

GU706831.1 Cydia pomonella voucher BC ZSM Lep 23607 cytochrome oxidase subunit 1 (COI) gene, partial cds; mitochondrial

GU706832.1 Cydia pomonella voucher BC ZSM Lep 23583 cytochrome oxidase subunit 1 (COI) gene, partial cds; mitochondrial

GU707060.1 Cydia pomonella voucher BC ZSM Lep 25712 cytochrome oxidase subunit 1 (COI) gene, partial cds; mitochondrial

GU800247.1 Cydia pomonella voucher BIOUG<CAN_:MDOK-1834 cytochrome oxidase subunit 1 (COI) gene, partial cds; mitochondrial

HM871982.1 Cydia pomonella voucher MM03480 cytochrome oxidase subunit 1 (COI) gene, partial cds; mitochondrial

HM874287.1 Cydia pomonella voucher MM09453 cytochrome oxidase subunit 1 (COI) gene, partial cds; mitochondrial

HM870894.1 Cydia pomonella voucher MM01761 cytochrome oxidase subunit 1 (COI) gene, partial cds; mitochondrial

HQ700336.1 Cydia pomonella voucher Mai03-256 cytochrome oxidase subunit I (COI) gene, partial cds; mitochondrial

HQ700337.1 Cydia pomonella voucher Mai03-180 cytochrome oxidase subunit I (COI) gene, partial cds; mitochondrial

JF859814.1 Cydia pomonella voucher TLMF Lep 02234 cytochrome oxidase subunit 1 (COI) gene, partial cds; mitochondrial

JF707773.1 Cydia pomonella voucher cp-001 cytochrome oxidase subunit I (COI) gene, partial cds; mitochondrial

KC120772.1 Cydia pomonella isolate TL-NKU12 cytochrome oxidase subunit 1 (COI) gene, partial cds; mitochondrial

KF397082.1 Cydia pomonella voucher 11ANIC-12889 cytochrome oxidase subunit 1 (COI) gene, partial cds; mitochondrial

KF405693.1 Cydia pomonella voucher 11ANIC-12891 cytochrome oxidase subunit 1 (COI) gene, partial cds; mitochondrial

KC315448.1 Cydia pomonella cytochrome c oxidase subunit I (COI) gene, partial cds; mitochondrial

KF491664.1 Cydia pomonella voucher JG-96-0004 cytochrome oxidase subunit 1 (COI) gene, partial cds; mitochondrial

KJ833877.1 Cydia pomonella voucher lxy3110101 cytochrome oxidase subunit I (COI) gene, partial cds; mitochondrial

KJ833878.1 Cydia pomonella voucher lxy3110102 cytochrome oxidase subunit I (COI) gene, partial cds; mitochondrial

KJ833879.1 Cydia pomonella voucher lxy3110103 cytochrome oxidase subunit I (COI) gene, partial cds; mitochondrial

KJ833880.1 Cydia pomonella voucher lxy3110104 cytochrome oxidase subunit I (COI) gene, partial cds; mitochondrial

KJ833881.1 Cydia pomonella voucher lxy3110105 cytochrome oxidase subunit I (COI) gene, partial cds; mitochondrial

KJ833882.1 Cydia pomonella voucher lxy3110106 cytochrome oxidase subunit I (COI) gene, partial cds; mitochondrial

KJ833883.1 Cydia pomonella voucher lxy3110107 cytochrome oxidase subunit I (COI) gene, partial cds; mitochondrial

KJ833884.1 Cydia pomonella voucher lxy3110108 cytochrome oxidase subunit I (COI) gene, partial cds; mitochondrial

KJ833885.1 Cydia pomonella voucher lxy3110109 cytochrome oxidase subunit I (COI) gene, partial cds; mitochondrial

KJ833886.1 Cydia pomonella voucher lxy3110110 cytochrome oxidase subunit I (COI) gene, partial cds; mitochondrial

KJ833887.1 Cydia pomonella voucher lxy3110111 cytochrome oxidase subunit I (COI) gene, partial cds; mitochondrial

KP072615.1 Cydia pomonella voucher Avi02-3 cytochrome oxidase subunit I (COI) gene, partial cds; mitochondrial

KP072616.1 Cydia pomonella voucher Val03-8 cytochrome oxidase subunit I (COI) gene, partial cds; mitochondrial

KP072617.1 Cydia pomonella voucher Val03-340 cytochrome oxidase subunit I (COI) gene, partial cds; mitochondrial

KP072618.1 Cydia pomonella voucher Mai03-256 cytochrome oxidase subunit I (COI) gene, partial cds; mitochondrial

KP072619.1 Cydia pomonella voucher Mai03-180 cytochrome oxidase subunit I (COI) gene, partial cds; mitochondrial

KP072620.1 Cydia pomonella voucher Tch05-43 cytochrome oxidase subunit I (COI) gene, partial cds; mitochondrial

KP072621.1 Cydia pomonella voucher Tch05-57 cytochrome oxidase subunit I (COI) gene, partial cds; mitochondrial

KP072622.1 Cydia pomonella voucher Tch05-94 cytochrome oxidase subunit I (COI) gene, partial cds; mitochondrial

KP072623.1 Cydia pomonella voucher Pc03-26 cytochrome oxidase subunit I (COI) gene, partial cds; mitochondrial

KP072624.1 Cydia pomonella voucher Pm03-24 cytochrome oxidase subunit I (COI) gene, partial cds; mitochondrial

KM572180.1 Cydia pomonella voucher TLMF Lep 07926 cytochrome oxidase subunit 1 (COI) gene, partial cds; mitochondrial

KR704270.1 Cydia pomonella isolate Cp-E-6 cytochrome oxidase subunit I (COI) gene, partial cds; mitochondrial

KR704271.1 Cydia pomonella isolate Cp-egg cytochrome oxidase subunit I (COI) gene, partial cds; mitochondrial

KX043809.1 Cydia pomonella voucher UKLB8F11 cytochrome oxidase subunit 1 (COI) gene, partial cds; mitochondrial

KX044056.1 Cydia pomonella voucher UKLB40G06 cytochrome oxidase subunit 1 (COI) gene, partial cds; mitochondrial

KX047884.1 Cydia pomonella voucher NHMO-07074 cytochrome oxidase subunit 1 (COI) gene, partial cds; mitochondrial

KT127215.1 Cydia pomonella voucher MNBTT-1571 cytochrome oxidase subunit 1 (COI) gene, partial cds; mitochondrial

KT128638.1 Cydia pomonella voucher PPBP-2177 cytochrome oxidase subunit 1 (COI) gene, partial cds; mitochondrial

KT131751.1 Cydia pomonella voucher MNBTT-1566 cytochrome oxidase subunit 1 (COI) gene, partial cds; mitochondrial

KT132572.1 Cydia pomonella voucher 2006-ONT-1028 cytochrome oxidase subunit 1 (COI) gene, partial cds; mitochondrial

KT133040.1 Cydia pomonella voucher MNBTT-1628 cytochrome oxidase subunit 1 (COI) gene, partial cds; mitochondrial

KT133122.1 Cydia pomonella voucher 2006-ONT-1026 cytochrome oxidase subunit 1 (COI) gene, partial cds; mitochondrial

KT133218.1 Cydia pomonella voucher PPBP-2179 cytochrome oxidase subunit 1 (COI) gene, partial cds; mitochondrial

KT137572.1 Cydia pomonella voucher MNBTT-1485 cytochrome oxidase subunit 1 (COI) gene, partial cds; mitochondrial

KT137944.1 Cydia pomonella voucher MNBTT-1627 cytochrome oxidase subunit 1 (COI) gene, partial cds; mitochondrial

KT142271.1 Cydia pomonella voucher PPBP-2185 cytochrome oxidase subunit 1 (COI) gene, partial cds; mitochondrial

KT144848.1 Cydia pomonella voucher 2006-ONT-1075 cytochrome oxidase subunit 1 (COI) gene, partial cds; mitochondrial

KT145660.1 Cydia pomonella voucher 2006-COFM-039 cytochrome oxidase subunit 1 (COI) gene, partial cds; mitochondrial

KT146697.1 Cydia pomonella voucher PPBP-2436 cytochrome oxidase subunit 1 (COI) gene, partial cds; mitochondrial

KT148062.1 Cydia pomonella voucher PPBP-2435 cytochrome oxidase subunit 1 (COI) gene, partial cds; mitochondrial

KT148382.1 Cydia pomonella voucher MNBTT-2321 cytochrome oxidase subunit 1 (COI) gene, partial cds; mitochondrial

MN122336.1 Cydia pomonella voucher 2017-Canakkale-Bayramic-1 cytochrome c oxidase subunit I gene, partial cds; mitochondrial

MN122337.1 Cydia pomonella voucher 2017-Canakkale-Bayramic-2 cytochrome c oxidase subunit I gene, partial cds; mitochondrial

MN122338.1 Cydia pomonella voucher 2017-Canakkale-Bayramic-3 cytochrome c oxidase subunit I gene, partial cds; mitochondrial

MN122339.1 Cydia pomonella voucher 2017-Canakkale-Bayramic-4 cytochrome c oxidase subunit I gene, partial cds; mitochondrial

MN122340.1 Cydia pomonella voucher 2017-Canakkale-Bayramic-5 cytochrome c oxidase subunit I gene, partial cds; mitochondrial

MN122341.1 Cydia pomonella voucher 2017-Canakkale-Bayramic-6 cytochrome c oxidase subunit I gene, partial cds; mitochondrial

MN122342.1 Cydia pomonella voucher 2017-Canakkale-Bayramic-7 cytochrome c oxidase subunit I gene, partial cds; mitochondrial

MN122343.1 Cydia pomonella voucher 2017-Canakkale-Bayramic-8 cytochrome c oxidase subunit I gene, partial cds; mitochondrial

MN122344.1 Cydia pomonella voucher 2017-Canakkale-Bayramic-9 cytochrome c oxidase subunit I gene, partial cds; mitochondrial

MN122345.1 Cydia pomonella voucher 2017-Canakkale-Ezine-1 cytochrome c oxidase subunit I gene, partial cds; mitochondrial

MN122346.1 Cydia pomonella voucher 2017-Canakkale-Ezine-2 cytochrome c oxidase subunit I gene, partial cds; mitochondrial

MN122347.1 Cydia pomonella voucher 2017-Canakkale-Ezine-3 cytochrome c oxidase subunit I gene, partial cds; mitochondrial

MN122348.1 Cydia pomonella voucher 2017-Canakkale-Ayvacik-1 cytochrome c oxidase subunit I gene, partial cds; mitochondrial

MN122349.1 Cydia pomonella voucher 2017-Canakkale-Ayvacik-2 cytochrome c oxidase subunit I gene, partial cds; mitochondrial

MN122350.1 Cydia pomonella voucher 2017-Canakkale-Ayvacik-3 cytochrome c oxidase subunit I gene, partial cds; mitochondrial

MN122351.1 Cydia pomonella voucher 2017-Canakkale-Bozcaada-1 cytochrome c oxidase subunit I gene, partial cds; mitochondrial

MN122352.1 Cydia pomonella voucher 2017-Canakkale-Bozcaada-2 cytochrome c oxidase subunit I gene, partial cds; mitochondrial

MN122353.1 Cydia pomonella voucher 2017-Canakkale-Bozcaada-3 cytochrome c oxidase subunit I gene, partial cds; mitochondrial

MN122354.1 Cydia pomonella voucher 2017-Canakkale-Center-1 cytochrome c oxidase subunit I gene, partial cds; mitochondrial

MN122355.1 Cydia pomonella voucher 2017-Canakkale-Center-2 cytochrome c oxidase subunit I gene, partial cds; mitochondrial

MN122356.1 Cydia pomonella voucher 2017-Canakkale-Center-3 cytochrome c oxidase subunit I gene, partial cds; mitochondrial

MN122357.1 Cydia pomonella voucher 2017-Canakkale-Dardanos-1 cytochrome c oxidase subunit I gene, partial cds; mitochondrial

MN122358.1 Cydia pomonella voucher 2017-Canakkale-Dardanos-2 cytochrome c oxidase subunit I gene, partial cds; mitochondrial

MN122359.1 Cydia pomonella voucher 2017-Canakkale-Dardanos-3 cytochrome c oxidase subunit I gene, partial cds; mitochondrial

MN122360.1 Cydia pomonella voucher 2017-Canakkale-Gokceada-1 cytochrome c oxidase subunit I gene, partial cds; mitochondrial

MN122361.1 Cydia pomonella voucher 2017-Canakkale-Gokceada-2 cytochrome c oxidase subunit I gene, partial cds; mitochondrial

MN122362.1 Cydia pomonella voucher 2017-Canakkale-Gokceada-3 cytochrome c oxidase subunit I gene, partial cds; mitochondrial

MN122363.1 Cydia pomonella voucher 2017-Canakkale-Lapseki-1 cytochrome c oxidase subunit I gene, partial cds; mitochondrial

MN122364.1 Cydia pomonella voucher 2017-Canakkale-Lapseki-2 cytochrome c oxidase subunit I gene, partial cds; mitochondrial

MN122365.1 Cydia pomonella voucher 2017-Canakkale-Lapseki-3 cytochrome c oxidase subunit I gene, partial cds; mitochondrial

MN122366.1 Cydia pomonella voucher 2017-Canakkale-Lapseki-4 cytochrome c oxidase subunit I gene, partial cds; mitochondrial

MN122367.1 Cydia pomonella voucher 2017-Canakkale-Lapseki-5 cytochrome c oxidase subunit I gene, partial cds; mitochondrial

MN122368.1 Cydia pomonella voucher 2017-Canakkale-Lapseki-6 cytochrome c oxidase subunit I gene, partial cds; mitochondrial

MN122369.1 Cydia pomonella voucher 2017-Canakkale-Eceabat-1 cytochrome c oxidase subunit I gene, partial cds; mitochondrial

MN122370.1 Cydia pomonella voucher 2017-Canakkale-Biga-1 cytochrome c oxidase subunit I gene, partial cds; mitochondrial

MN122371.1 Cydia pomonella voucher 2017-Canakkale-Biga-2 cytochrome c oxidase subunit I gene, partial cds; mitochondrial

MN122372.1 Cydia pomonella voucher 2017-Balikesir-Center-1 cytochrome c oxidase subunit I gene, partial cds; mitochondrial

MN122373.1 Cydia pomonella voucher 2017-Balikesir-Center-2 cytochrome c oxidase subunit I gene, partial cds; mitochondrial

MN122374.1 Cydia pomonella voucher 2017-Balikesir-Center-3 cytochrome c oxidase subunit I gene, partial cds; mitochondrial

MN122375.1 Cydia pomonella voucher 2017-Bursa-Orhaneli-1 cytochrome c oxidase subunit I gene, partial cds; mitochondrial

MN122376.1 Cydia pomonella voucher 2017-Bursa-Orhaneli-2 cytochrome c oxidase subunit I gene, partial cds; mitochondrial

MN122377.1 Cydia pomonella voucher 2017-Bursa-Orhaneli-3 cytochrome c oxidase subunit I gene, partial cds; mitochondrial

MN122378.1 Cydia pomonella voucher 2017-Bursa-Inegol-1 cytochrome c oxidase subunit I gene, partial cds; mitochondrial

MN122379.1 Cydia pomonella voucher 2017-Bursa-Inegol-2 cytochrome c oxidase subunit I gene, partial cds; mitochondrial

MN122380.1 Cydia pomonella voucher 2017-Bursa-Inegol-3 cytochrome c oxidase subunit I gene, partial cds; mitochondrial

MN122381.1 Cydia pomonella voucher 2017-Istanbul-Tuzla-1 cytochrome c oxidase subunit I gene, partial cds; mitochondrial

MN122382.1 Cydia pomonella voucher 2017-Istanbul-Tuzla-2 cytochrome c oxidase subunit I gene, partial cds; mitochondrial

MN122383.1 Cydia pomonella voucher 2017-Istanbul-Tuzla-3 cytochrome c oxidase subunit I gene, partial cds; mitochondrial

MN122384.1 Cydia pomonella voucher 2017-Izmir-Torbali-1 cytochrome c oxidase subunit I gene, partial cds; mitochondrial

MN122385.1 Cydia pomonella voucher 2017-Izmir-Torbali-2 cytochrome c oxidase subunit I gene, partial cds; mitochondrial

MN122386.1 Cydia pomonella voucher 2017-Izmir-Torbali-3 cytochrome c oxidase subunit I gene, partial cds; mitochondrial

MN122387.1 Cydia pomonella voucher 2017-Ordu-Fatsa-1 cytochrome c oxidase subunit I gene, partial cds; mitochondrial

MN122388.1 Cydia pomonella voucher 2017-Ordu-Fatsa-2 cytochrome c oxidase subunit I gene, partial cds; mitochondrial

MN122389.1 Cydia pomonella voucher 2017-Ordu-Fatsa-3 cytochrome c oxidase subunit I gene, partial cds; mitochondrial

MN122390.1 Cydia pomonella voucher 2017-Erzurum-Tortum-1 cytochrome c oxidase subunit I gene, partial cds; mitochondrial

MN122391.1 Cydia pomonella voucher 2017-Erzurum-Tortum-2 cytochrome c oxidase subunit I gene, partial cds; mitochondrial

MN122392.1 Cydia pomonella voucher 2017-Erzurum-Tortum-3 cytochrome c oxidase subunit I gene, partial cds; mitochondrial

MN122393.1 Cydia pomonella voucher 2017-Adana-Pozanti-1 cytochrome c oxidase subunit I gene, partial cds; mitochondrial

MN122394.1 Cydia pomonella voucher 2017-Adana-Pozanti-2 cytochrome c oxidase subunit I gene, partial cds; mitochondrial

MN122395.1 Cydia pomonella voucher 2017-Van-Muradiye-1 cytochrome c oxidase subunit I gene, partial cds; mitochondrial

MN122396.1 Cydia pomonella voucher 2017-Van-Muradiye-2 cytochrome c oxidase subunit I gene, partial cds; mitochondrial

MK759920.1 Cydia pomonella isolate S1 cytochrome oxidase subunit I (COI) gene, partial cds; mitochondrial

MK759921.1 Cydia pomonella isolate S2 cytochrome oxidase subunit I (COI) gene, partial cds; mitochondrial

MK759922.1 Cydia pomonella isolate M1 cytochrome oxidase subunit I (COI) gene, partial cds; mitochondrial

MK759923.1 Cydia pomonella isolate M2 cytochrome oxidase subunit I (COI) gene, partial cds; mitochondrial

MK759926.1 Cydia pomonella isolate K1 cytochrome oxidase subunit I (COI) gene, partial cds; mitochondrial

MK759927.1 Cydia pomonella isolate K2 cytochrome oxidase subunit I (COI) gene, partial cds; mitochondrial

MK732894.1 Cydia pomonella isolate Ka1Khaltsi cytochrome oxidase subunit I (COI) gene, partial cds; mitochondrial

MK732895.1 Cydia pomonella isolate Ka2Khaltsi cytochrome oxidase subunit I (COI) gene, partial cds; mitochondrial

MK732896.1 Cydia pomonella isolate Sa1Sanjak cytochrome oxidase subunit I (COI) gene, partial cds; mitochondrial

MK732897.1 Cydia pomonella isolate Sa2Sanjak cytochrome oxidase subunit I (COI) gene, partial cds; mitochondrial

MK732898.1 Cydia pomonella isolate N1Nimo cytochrome oxidase subunit I (COI) gene, partial cds; mitochondrial

MK732899.1 Cydia pomonella isolate N2Nimo cytochrome oxidase subunit I (COI) gene, partial cds; mitochondrial

MK759928.1 Cydia pomonella isolate O1Oley cytochrome oxidase subunit I (COI) gene, partial cds; mitochondrial

MK759929.1 Cydia pomonella isolate O2Oley cytochrome oxidase subunit I (COI) gene, partial cds; mitochondrial

MK759930.1 Cydia pomonella isolate B1Basgo cytochrome oxidase subunit I (COI) gene, partial cds; mitochondrial

MK759931.1 Cydia pomonella isolate B2Basgo cytochrome oxidase subunit I (COI) gene, partial cds; mitochondrial

MK759932.1 Cydia pomonella isolate G1Garkone cytochrome oxidase subunit I (COI) gene, partial cds; mitochondrial

MK759933.1 Cydia pomonella isolate G2Garkone cytochrome oxidase subunit I (COI) gene, partial cds; mitochondrial

MK759934.1 Cydia pomonella isolate Th1Thiksei cytochrome oxidase subunit I (COI) gene, partial cds; mitochondrial

MK759935.1 Cydia pomonella isolate Th2Thiksei cytochrome oxidase subunit I (COI) gene, partial cds; mitochondrial

MK759936.1 Cydia pomonella isolate Ga1Gargarthla cytochrome oxidase subunit I (COI) gene, partial cds; mitochondrial

MK759937.1 Cydia pomonella isolate Ga2Gargarthla cytochrome oxidase subunit I (COI) gene, partial cds; mitochondrial

MK759938.1 Cydia pomonella isolate AK1Akchamal cytochrome oxidase subunit I (COI) gene, partial cds; mitochondrial

MK759939.1 Cydia pomonella isolate AK2Akchamal cytochrome oxidase subunit I (COI) gene, partial cds; mitochondrial

MK759940.1 Cydia pomonella isolate Dh1Dha cytochrome oxidase subunit I (COI) gene, partial cds; mitochondrial

MK759941.1 Cydia pomonella isolate Dh2Dha cytochrome oxidase subunit I (COI) gene, partial cds; mitochondrial

MK759944.1 Cydia pomonella isolate Sh1Shspol cytochrome oxidase subunit I (COI) gene, partial cds; mitochondrial

MK759945.1 Cydia pomonella isolate Sh2Shspol cytochrome oxidase subunit I (COI) gene, partial cds; mitochondrial

MK759946.1 Cydia pomonella isolate LH1Leh cytochrome oxidase subunit I (COI) gene, partial cds; mitochondrial

MK759947.1 Cydia pomonella isolate LH2Leh cytochrome oxidase subunit I (COI) gene, partial cds; mitochondrial

MN972450.1 Cydia pomonella voucher CIMA-INSECT-098 cytochrome oxidase subunit I (COI) gene, partial cds; mitochondrial

MW269982.1 Cydia pomonella isolate KA03 cytochrome c oxidase subunit I (COX1) gene, partial cds; mitochondrial

MW269983.1 Cydia pomonella isolate KA13 cytochrome c oxidase subunit I (COX1) gene, partial cds; mitochondrial

MW269984.1 Cydia pomonella isolate KA01 cytochrome c oxidase subunit I (COX1) gene, partial cds; mitochondrial

MW269985.1 Cydia pomonella isolate KA07 cytochrome c oxidase subunit I (COX1) gene, partial cds; mitochondrial

MW269986.1 Cydia pomonella isolate KA09 cytochrome c oxidase subunit I (COX1) gene, partial cds; mitochondrial

MW269987.1 Cydia pomonella isolate KA11 cytochrome c oxidase subunit I (COX1) gene, partial cds; mitochondrial

MW269988.1 Cydia pomonella isolate BO03 cytochrome c oxidase subunit I (COX1) gene, partial cds; mitochondrial

MW269989.1 Cydia pomonella isolate BO07 cytochrome c oxidase subunit I (COX1) gene, partial cds; mitochondrial

MW269990.1 Cydia pomonella isolate BO09 cytochrome c oxidase subunit I (COX1) gene, partial cds; mitochondrial

MW269991.1 Cydia pomonella isolate BO11 cytochrome c oxidase subunit I (COX1) gene, partial cds; mitochondrial

MW269992.1 Cydia pomonella isolate DH11 cytochrome c oxidase subunit I (COX1) gene, partial cds; mitochondrial

MW269993.1 Cydia pomonella isolate DH09 cytochrome c oxidase subunit I (COX1) gene, partial cds; mitochondrial

MW269994.1 Cydia pomonella isolate DH07 cytochrome c oxidase subunit I (COX1) gene, partial cds; mitochondrial

MW269995.1 Cydia pomonella isolate DH05 cytochrome c oxidase subunit I (COX1) gene, partial cds; mitochondrial

MW269996.1 Cydia pomonella isolate DH03 cytochrome c oxidase subunit I (COX1) gene, partial cds; mitochondrial

MW269997.1 Cydia pomonella isolate DH01 cytochrome c oxidase subunit I (COX1) gene, partial cds; mitochondrial

MW269998.1 Cydia pomonella isolate GA03 cytochrome c oxidase subunit I (COX1) gene, partial cds; mitochondrial

MW269999.1 Cydia pomonella isolate GA05 cytochrome c oxidase subunit I (COX1) gene, partial cds; mitochondrial

MW270000.1 Cydia pomonella isolate GA07 cytochrome c oxidase subunit I (COX1) gene, partial cds; mitochondrial

MW270001.1 Cydia pomonella isolate GA09 cytochrome c oxidase subunit I (COX1) gene, partial cds; mitochondrial

MW270002.1 Cydia pomonella isolate GA11 cytochrome c oxidase subunit I (COX1) gene, partial cds; mitochondrial

MW270003.1 Cydia pomonella isolate GG11 cytochrome c oxidase subunit I (COX1) gene, partial cds; mitochondrial

MW270004.1 Cydia pomonella isolate GG09 cytochrome c oxidase subunit I (COX1) gene, partial cds; mitochondrial

MW270005.1 Cydia pomonella isolate GG07 cytochrome c oxidase subunit I (COX1) gene, partial cds; mitochondrial

MW270006.1 Cydia pomonella isolate GG05 cytochrome c oxidase subunit I (COX1) gene, partial cds; mitochondrial

MW270007.1 Cydia pomonella isolate GG03 cytochrome c oxidase subunit I (COX1) gene, partial cds; mitochondrial

MW270008.1 Cydia pomonella isolate GG01 cytochrome c oxidase subunit I (COX1) gene, partial cds; mitochondrial

MW270009.1 Cydia pomonella isolate KR01 cytochrome c oxidase subunit I (COX1) gene, partial cds; mitochondrial

MW270010.1 Cydia pomonella isolate KR07 cytochrome c oxidase subunit I (COX1) gene, partial cds; mitochondrial

MW270011.1 Cydia pomonella isolate MI01 cytochrome c oxidase subunit I (COX1) gene, partial cds; mitochondrial

MW270012.1 Cydia pomonella isolate MI03 cytochrome c oxidase subunit I (COX1) gene, partial cds; mitochondrial

MW270013.1 Cydia pomonella isolate MI05 cytochrome c oxidase subunit I (COX1) gene, partial cds; mitochondrial

MW270014.1 Cydia pomonella isolate MI08 cytochrome c oxidase subunit I (COX1) gene, partial cds; mitochondrial

MW270015.1 Cydia pomonella isolate MI09 cytochrome c oxidase subunit I (COX1) gene, partial cds; mitochondrial

MW270016.1 Cydia pomonella isolate MI11 cytochrome c oxidase subunit I (COX1) gene, partial cds; mitochondrial

MW270017.1 Cydia pomonella isolate NI11 cytochrome c oxidase subunit I (COX1) gene, partial cds; mitochondrial

MW270018.1 Cydia pomonella isolate NI13 cytochrome c oxidase subunit I (COX1) gene, partial cds; mitochondrial

MW270019.1 Cydia pomonella isolate NI09 cytochrome c oxidase subunit I (COX1) gene, partial cds; mitochondrial

MW270020.1 Cydia pomonella isolate NI07 cytochrome c oxidase subunit I (COX1) gene, partial cds; mitochondrial

MW270021.1 Cydia pomonella isolate NI03 cytochrome c oxidase subunit I (COX1) gene, partial cds; mitochondrial

MW270022.1 Cydia pomonella isolate NI01 cytochrome c oxidase subunit I (COX1) gene, partial cds; mitochondrial

MW270023.1 Cydia pomonella isolate OL01 cytochrome c oxidase subunit I (COX1) gene, partial cds; mitochondrial

MW270024.1 Cydia pomonella isolate OL03 cytochrome c oxidase subunit I (COX1) gene, partial cds; mitochondrial

MW270025.1 Cydia pomonella isolate OL05 cytochrome c oxidase subunit I (COX1) gene, partial cds; mitochondrial

MW270026.1 Cydia pomonella isolate OL07 cytochrome c oxidase subunit I (COX1) gene, partial cds; mitochondrial

MW270027.1 Cydia pomonella isolate OL09 cytochrome c oxidase subunit I (COX1) gene, partial cds; mitochondrial

MW270028.1 Cydia pomonella isolate OL11 cytochrome c oxidase subunit I (COX1) gene, partial cds; mitochondrial

MW270029.1 Cydia pomonella isolate SA09 cytochrome c oxidase subunit I (COX1) gene, partial cds; mitochondrial

MW270030.1 Cydia pomonella isolate SA07 cytochrome c oxidase subunit I (COX1) gene, partial cds; mitochondrial

MW270031.1 Cydia pomonella isolate SA01 cytochrome c oxidase subunit I (COX1) gene, partial cds; mitochondrial

MW270032.1 Cydia pomonella isolate SA11 cytochrome c oxidase subunit I (COX1) gene, partial cds; mitochondrial

MW270033.1 Cydia pomonella isolate SA05 cytochrome c oxidase subunit I (COX1) gene, partial cds; mitochondrial

MW270034.1 Cydia pomonella isolate SA03 cytochrome c oxidase subunit I (COX1) gene, partial cds; mitochondrial

MW270035.1 Cydia pomonella isolate SS01 cytochrome c oxidase subunit I (COX1) gene, partial cds; mitochondrial

MW270036.1 Cydia pomonella isolate SS03 cytochrome c oxidase subunit I (COX1) gene, partial cds; mitochondrial

MW270037.1 Cydia pomonella isolate SS09 cytochrome c oxidase subunit I (COX1) gene, partial cds; mitochondrial

MW270038.1 Cydia pomonella isolate SH03 cytochrome c oxidase subunit I (COX1) gene, partial cds; mitochondrial

MW270039.1 Cydia pomonella isolate SH04 cytochrome c oxidase subunit I (COX1) gene, partial cds; mitochondrial

MW270040.1 Cydia pomonella isolate SH06 cytochrome c oxidase subunit I (COX1) gene, partial cds; mitochondrial

MW270041.1 Cydia pomonella isolate SH08 cytochrome c oxidase subunit I (COX1) gene, partial cds; mitochondrial

MW270042.1 Cydia pomonella isolate SH12 cytochrome c oxidase subunit I (COX1) gene, partial cds; mitochondrial

MW270043.1 Cydia pomonella isolate TH03 cytochrome c oxidase subunit I (COX1) gene, partial cds; mitochondrial

MW270044.1 Cydia pomonella isolate TH04 cytochrome c oxidase subunit I (COX1) gene, partial cds; mitochondrial

MW270045.1 Cydia pomonella isolate TH10 cytochrome c oxidase subunit I (COX1) gene, partial cds; mitochondrial

MW270046.1 Cydia pomonella isolate TH12 cytochrome c oxidase subunit I (COX1) gene, partial cds; mitochondrial

MW270047.1 Cydia pomonella isolate TH06 cytochrome c oxidase subunit I (COX1) gene, partial cds; mitochondrial

MW270048.1 Cydia pomonella isolate TH08 cytochrome c oxidase subunit I (COX1) gene, partial cds; mitochondrial

MW270049.1 Cydia pomonella isolate AK03 cytochrome c oxidase subunit I (COX1) gene, partial cds; mitochondrial

MW270050.1 Cydia pomonella isolate AK05 cytochrome c oxidase subunit I (COX1) gene, partial cds; mitochondrial

MW270051.1 Cydia pomonella isolate AK07 cytochrome c oxidase subunit I (COX1) gene, partial cds; mitochondrial

MW270052.1 Cydia pomonella isolate AK09 cytochrome c oxidase subunit I (COX1) gene, partial cds; mitochondrial

MW270053.1 Cydia pomonella isolate AK11 cytochrome c oxidase subunit I (COX1) gene, partial cds; mitochondrial

MW270054.1 Cydia pomonella isolate AK13 cytochrome c oxidase subunit I (COX1) gene, partial cds; mitochondrial

MW270055.1 Cydia pomonella isolate HN03 cytochrome c oxidase subunit I (COX1) gene, partial cds; mitochondrial

MW270056.1 Cydia pomonella isolate HN09 cytochrome c oxidase subunit I (COX1) gene, partial cds; mitochondrial

MW270057.1 Cydia pomonella isolate HN11 cytochrome c oxidase subunit I (COX1) gene, partial cds; mitochondrial

MW270058.1 Cydia pomonella isolate HN13 cytochrome c oxidase subunit I (COX1) gene, partial cds; mitochondrial

MW270059.1 Cydia pomonella isolate LH01 cytochrome c oxidase subunit I (COX1) gene, partial cds; mitochondrial

MW270060.1 Cydia pomonella isolate LH03 cytochrome c oxidase subunit I (COX1) gene, partial cds; mitochondrial

MW270061.1 Cydia pomonella isolate LH05 cytochrome c oxidase subunit I (COX1) gene, partial cds; mitochondrial

MW270062.1 Cydia pomonella isolate LH07 cytochrome c oxidase subunit I (COX1) gene, partial cds; mitochondrial

MW270063.1 Cydia pomonella isolate LH10 cytochrome c oxidase subunit I (COX1) gene, partial cds; mitochondrial

MW270064.1 Cydia pomonella isolate LH11 cytochrome c oxidase subunit I (COX1) gene, partial cds; mitochondrial

MW270065.1 Cydia pomonella isolate HA09 cytochrome c oxidase subunit I (COX1) gene, partial cds; mitochondrial

MW270066.1 Cydia pomonella isolate HA05 cytochrome c oxidase subunit I (COX1) gene, partial cds; mitochondrial

MW270067.1 Cydia pomonella isolate HA08 cytochrome c oxidase subunit I (COX1) gene, partial cds; mitochondrial

MW380949.1 Cydia pomonella strain Nadihal Kashmir cytochrome c oxidase subunit I (COX1) gene, partial cds; mitochondrial

MW380950.1 Cydia pomonella strain Kargil Ladakh cytochrome c oxidase subunit I (COX1) gene, partial cds; mitochondrial

OQ186760.1 Cydia pomonella isolate A-4 cytochrome c oxidase subunit I (COX1) gene, partial cds; mitochondrial

OQ186859.1 Cydia pomonella isolate A-3 cytochrome c oxidase subunit I (COX1) gene, partial cds; mitochondrial

OQ187770.1 Cydia pomonella isolate A-1 cytochrome c oxidase subunit I (COX1) gene, partial cds; mitochondrial

OQ190153.1 Cydia pomonella isolate A-2 cytochrome c oxidase subunit I (COX1) gene, partial cds; mitochondrial

OQ306612.1 Cydia pomonella isolate CD1 cytochrome c oxidase subunit I (COX1) gene, partial cds; mitochondrial

OQ308608.1 Cydia pomonella isolate CD2 cytochrome c oxidase subunit I (COX1) gene, partial cds; mitochondrial

OQ836333.1 Cydia pomonella voucher 1842082 cytochrome c oxidase subunit I (COX1) gene, partial cds; mitochondrial

OQ836340.1 Cydia pomonella voucher 32550162 cytochrome c oxidase subunit I (COX1) gene, partial cds; mitochondrial

OQ836341.1 Cydia pomonella voucher 32904311 cytochrome c oxidase subunit I (COX1) gene, partial cds; mitochondrial

OQ836342.1 Cydia pomonella voucher 33362251 cytochrome c oxidase subunit I (COX1) gene, partial cds; mitochondrial

OQ836343.1 Cydia pomonella voucher 36812203 cytochrome c oxidase subunit I (COX1) gene, partial cds; mitochondrial

OQ836346.1 Cydia pomonella voucher 36300726A cytochrome c oxidase subunit I (COX1) gene, partial cds; mitochondrial

OQ836351.1 Cydia pomonella voucher 256565 cytochrome c oxidase subunit I (COX1) gene, partial cds; mitochondrial

OQ836372.1 Cydia pomonella voucher JWB-09-0038 cytochrome c oxidase subunit I (COX1) gene, partial cds; mitochondrial

OQ836402.1 Cydia pomonella voucher TOR-DNA-1131 cytochrome c oxidase subunit I (COX1) gene, partial cds; mitochondrial

OQ836403.1 Cydia pomonella voucher TOR-DNA-1167 cytochrome c oxidase subunit I (COX1) gene, partial cds; mitochondrial

OQ836404.1 Cydia pomonella voucher TOR-DNA-1193 cytochrome c oxidase subunit I (COX1) gene, partial cds; mitochondrial

OQ836405.1 Cydia pomonella voucher TOR-DNA-1204 cytochrome c oxidase subunit I (COX1) gene, partial cds; mitochondrial

OQ836406.1 Cydia pomonella voucher TOR-DNA-1216 cytochrome c oxidase subunit I (COX1) gene, partial cds; mitochondrial

OQ836407.1 Cydia pomonella voucher TOR-DNA-1226 cytochrome c oxidase subunit I (COX1) gene, partial cds; mitochondrial

OQ836408.1 Cydia pomonella voucher TOR-DNA-1260 cytochrome c oxidase subunit I (COX1) gene, partial cds; mitochondrial

OQ836409.1 Cydia pomonella voucher TOR-DNA-1287 cytochrome c oxidase subunit I (COX1) gene, partial cds; mitochondrial

OQ836410.1 Cydia pomonella voucher TOR-DNA-1300 cytochrome c oxidase subunit I (COX1) gene, partial cds; mitochondrial

OQ836411.1 Cydia pomonella voucher TOR-DNA-1335 cytochrome c oxidase subunit I (COX1) gene, partial cds; mitochondrial

OQ836412.1 Cydia pomonella voucher TOR-DNA-1341 cytochrome c oxidase subunit I (COX1) gene, partial cds; mitochondrial

OQ836413.1 Cydia pomonella voucher TOR-DNA-1351 cytochrome c oxidase subunit I (COX1) gene, partial cds; mitochondrial

OQ183111.1 Cydia pomonella voucher KLM Lep 15499 cytochrome oxidase subunit 1 (COI) gene, partial cds; mitochondrial

OQ562450.1 Cydia pomonella voucher INV00901 cytochrome oxidase subunit 1 (COI) gene, partial cds; mitochondrial

OQ563167.1 Cydia pomonella voucher INV05366 cytochrome oxidase subunit 1 (COI) gene, partial cds; mitochondrial

OQ564278.1 Cydia pomonella voucher INV06301 cytochrome oxidase subunit 1 (COI) gene, partial cds; mitochondrial

PP991409.1 Cydia pomonella isolate cp13 cytochrome c oxidase subunit I (COX1) gene, partial cds; mitochondrial

PQ072322.1 Cydia pomonella isolate Algeria_haplotype_10 cytochrome c oxidase subunit I (COX1) gene, partial cds; mitochondrial

PQ072323.1 Cydia pomonella isolate Algeria_haplotype_18 cytochrome c oxidase subunit I (COX1) gene, partial cds; mitochondrial

PQ072324.1 Cydia pomonella isolate Algeria_haplotype_19 cytochrome c oxidase subunit I (COX1) gene, partial cds; mitochondrial

PQ072325.1 Cydia pomonella isolate Algeria_haplotype_20 cytochrome c oxidase subunit I (COX1) gene, partial cds; mitochondrial

PQ072326.1 Cydia pomonella isolate Algeria_haplotype_22 cytochrome c oxidase subunit I (COX1) gene, partial cds; mitochondrial

PQ072327.1 Cydia pomonella isolate Algeria_haplotype_31 cytochrome c oxidase subunit I (COX1) gene, partial cds; mitochondrial

PQ072328.1 Cydia pomonella isolate Algeria_haplotype_7 cytochrome c oxidase subunit I (COX1) gene, partial cds; mitochondrial

PV412925.1 Cydia pomonella voucher Cp-RanchoNuevo cytochrome c oxidase subunit I (COX1) gene, partial cds; mitochondrial

PV707120.1 Cydia pomonella isolate 1 cytochrome c oxidase subunit I (COX1) gene, partial cds; mitochondrial

PV707121.1 Cydia pomonella isolate 2 cytochrome c oxidase subunit I (COX1) gene, partial cds; mitochondrial

PV707122.1 Cydia pomonella isolate 3 cytochrome c oxidase subunit I (COX1) gene, partial cds; mitochondrial

PV707123.1 Cydia pomonella isolate 4 cytochrome c oxidase subunit I (COX1) gene, partial cds; mitochondrial

PV707124.1 Cydia pomonella isolate 5 cytochrome c oxidase subunit I (COX1) gene, partial cds; mitochondrial

PV707125.1 Cydia pomonella isolate 6 cytochrome c oxidase subunit I (COX1) gene, partial cds; mitochondrial

PV707126.1 Cydia pomonella isolate 7 cytochrome c oxidase subunit I (COX1) gene, partial cds; mitochondrial

PV707127.1 Cydia pomonella isolate 8 cytochrome c oxidase subunit I (COX1) gene, partial cds; mitochondrial

PX471500.1 Cydia pomonella isolate Iraq-1 cytochrome c oxidase subunit I (COX1) gene, partial cds; mitochondrial

*Cydia splendana*

GU706830.1 Cydia splendana voucher BC ZSM Lep 23584 cytochrome oxidase subunit 1 (COI) gene, partial cds; mitochondrial

HM405789.1 Cydia splendana voucher UKLB40G05 cytochrome oxidase subunit 1 (COI) gene, partial cds; mitochondrial

HM875516.1 Cydia splendana voucher MM13175 cytochrome oxidase subunit 1 (COI) gene, partial cds; mitochondrial

HM871969.1 Cydia splendana voucher MM03458 cytochrome oxidase subunit 1 (COI) gene, partial cds; mitochondrial

HM872047.1 Cydia splendana voucher MM03565 cytochrome oxidase subunit 1 (COI) gene, partial cds; mitochondrial

HM874018.1 Cydia splendana voucher MM08633 cytochrome oxidase subunit 1 (COI) gene, partial cds; mitochondrial

JF859773.1 Cydia splendana voucher TLMF Lep 02190 cytochrome oxidase subunit 1 (COI) gene, partial cds; mitochondrial

KP072642.1 Cydia splendana voucher Div-09-6611 cytochrome oxidase subunit I (COI) gene, partial cds; mitochondrial

KP072643.1 Cydia splendana voucher Div-09-6599 cytochrome oxidase subunit I (COI) gene, partial cds; mitochondrial

KP072644.1 Cydia splendana voucher Div-09-6598 cytochrome oxidase subunit I (COI) gene, partial cds; mitochondrial

KP072645.1 Cydia splendana voucher Div-09-6612 cytochrome oxidase subunit I (COI) gene, partial cds; mitochondrial

KM572672.1 Cydia splendana voucher TLMF Lep 08032 cytochrome oxidase subunit 1 (COI) gene, partial cds; mitochondrial

KX046299.1 Cydia splendana voucher BC ZSM Lep 64325 cytochrome oxidase subunit 1 (COI) gene, partial cds; mitochondrial

KX046966.1 Cydia splendana voucher KBE-07012 cytochrome oxidase subunit 1 (COI) gene, partial cds; mitochondrial

KX042876.1 Cydia splendana voucher UKLB5B04 cytochrome oxidase subunit 1 (COI) gene, partial cds; mitochondrial

KX043638.1 Cydia splendana voucher UKLB34F12 cytochrome oxidase subunit 1 (COI) gene, partial cds; mitochondrial

KX048000.1 Cydia splendana voucher NHMO-07073 cytochrome oxidase subunit 1 (COI) gene, partial cds; mitochondrial

KX044910.1 Cydia splendana voucher BC ZSM Lep 64327 cytochrome oxidase subunit 1 (COI) gene, partial cds; mitochondrial

OQ706407.1 Cydia splendana voucher MM26719 cytochrome oxidase subunit 1 (COI) gene, partial cds; mitochondrial

OQ182901.1 Cydia splendana voucher TLMF_Lep_33361 cytochrome oxidase subunit 1 (COI) gene, partial cds; mitochondrial

OQ562903.1 Cydia splendana voucher INV03684 cytochrome oxidase subunit 1 (COI) gene, partial cds; mitochondrial

OQ563529.1 Cydia splendana voucher INV03627 cytochrome oxidase subunit 1 (COI) gene, partial cds; mitochondrial

OQ563698.1 Cydia splendana voucher INV01957 cytochrome oxidase subunit 1 (COI) gene, partial cds; mitochondrial

OQ563890.1 Cydia splendana voucher INV09810 cytochrome oxidase subunit 1 (COI) gene, partial cds; mitochondrial

*Grapholita funebrana*

GU706407.1 Grapholita funebrana voucher BC ZSM Lep 23325 cytochrome oxidase subunit 1 (COI) gene, partial cds; mitochondrial

GU706840.1 Grapholita funebrana voucher BC ZSM Lep 25306 cytochrome oxidase subunit 1 (COI) gene, partial cds; mitochondrial

GU706842.1 Grapholita funebrana voucher BC ZSM Lep 25506 cytochrome oxidase subunit 1 (COI) gene, partial cds; mitochondrial

HM876524.1 Grapholita funebrana voucher MM15743 cytochrome oxidase subunit 1 (COI) gene, partial cds; mitochondrial

HM873524.1 Grapholita funebrana voucher MM06833 cytochrome oxidase subunit 1 (COI) gene, partial cds; mitochondrial

HM873539.1 Grapholita funebrana voucher MM06851 cytochrome oxidase subunit 1 (COI) gene, partial cds; mitochondrial

KJ833896.1 Grapholita funebrana voucher lxy3110120 cytochrome oxidase subunit I (COI) gene, partial cds; mitochondrial

KJ833897.1 Grapholita funebrana voucher lxy3110121 cytochrome oxidase subunit I (COI) gene, partial cds; mitochondrial

KJ833898.1 Grapholita funebrana voucher lxy3110122 cytochrome oxidase subunit I (COI) gene, partial cds; mitochondrial

KJ833899.1 Grapholita funebrana voucher lxy3110123 cytochrome oxidase subunit I (COI) gene, partial cds; mitochondrial

KJ833900.1 Grapholita funebrana voucher lxy3110124 cytochrome oxidase subunit I (COI) gene, partial cds; mitochondrial

KJ833901.1 Grapholita funebrana voucher lxy3110125 cytochrome oxidase subunit I (COI) gene, partial cds; mitochondrial

KJ833902.1 Grapholita funebrana voucher lxy3110126 cytochrome oxidase subunit I (COI) gene, partial cds; mitochondrial

KJ833903.1 Grapholita funebrana voucher lxy3110127 cytochrome oxidase subunit I (COI) gene, partial cds; mitochondrial

KJ833904.1 Grapholita funebrana voucher lxy3110128 cytochrome oxidase subunit I (COI) gene, partial cds; mitochondrial

KJ833905.1 Grapholita funebrana voucher lxy3110129 cytochrome oxidase subunit I (COI) gene, partial cds; mitochondrial

KJ833906.1 Grapholita funebrana voucher lxy3110130 cytochrome oxidase subunit I (COI) gene, partial cds; mitochondrial

KJ833907.1 Grapholita funebrana voucher lxy3110131 cytochrome oxidase subunit I (COI) gene, partial cds; mitochondrial

KJ833908.1 Grapholita funebrana voucher lxy3110132 cytochrome oxidase subunit I (COI) gene, partial cds; mitochondrial

KJ833909.1 Grapholita funebrana voucher lxy3110133 cytochrome oxidase subunit I (COI) gene, partial cds; mitochondrial

KP072651.1 Grapholita funebrana voucher Div-13-12 cytochrome oxidase subunit I (COI) gene, partial cds; mitochondrial

KP072652.1 Grapholita funebrana voucher Div-13-15 cytochrome oxidase subunit I (COI) gene, partial cds; mitochondrial

KP072653.1 Grapholita funebrana voucher Div-13-16 cytochrome oxidase subunit I (COI) gene, partial cds; mitochondrial

KP072654.1 Grapholita funebrana voucher Div-13-17 cytochrome oxidase subunit I (COI) gene, partial cds; mitochondrial

KX230441.1 Grapholita funebrana voucher GEF1 cytochrome oxidase subunit 1 (COI) gene, partial cds; mitochondrial

KX230442.1 Grapholita funebrana voucher GEF2 cytochrome oxidase subunit 1 (COI) gene, partial cds; mitochondrial

KX230443.1 Grapholita funebrana voucher GEF3 cytochrome oxidase subunit 1 (COI) gene, partial cds; mitochondrial

KX230444.1 Grapholita funebrana voucher GF-SX-201301 cytochrome oxidase subunit 1 (COI) gene, partial cds; mitochondrial

KX230445.1 Grapholita funebrana voucher GF-SX-201305 cytochrome oxidase subunit 1 (COI) gene, partial cds; mitochondrial

KX230446.1 Grapholita funebrana voucher GF-SX-201308 cytochrome oxidase subunit 1 (COI) gene, partial cds; mitochondrial

KX260323.1 Grapholita funebrana isolate NL cytochrome oxidase subunit I (COI) gene, partial cds; mitochondrial

KX260324.1 Grapholita funebrana isolate CN cytochrome oxidase subunit I (COI) gene, partial cds; mitochondrial

KX046415.1 Grapholita funebrana voucher BC ZSM Lep 64331 cytochrome oxidase subunit 1 (COI) gene, partial cds; mitochondrial

KX043045.1 Grapholita funebrana voucher UKLB43A08 cytochrome oxidase subunit 1 (COI) gene, partial cds; mitochondrial

KX043273.1 Grapholita funebrana voucher UKLB34F09 cytochrome oxidase subunit 1 (COI) gene, partial cds; mitochondrial

KX047590.1 Grapholita funebrana voucher NHMO-06157 cytochrome oxidase subunit 1 (COI) gene, partial cds; mitochondrial

OL966296.1 Grapholita funebrana isolate fruit cytochrome c oxidase subunit I (COX1) gene, partial cds; mitochondrial

OQ836344.1 Grapholita funebrana voucher 4520337A cytochrome c oxidase subunit I (COX1) gene, partial cds; mitochondrial

OQ706476.1 Grapholita funebrana voucher MM26369 cytochrome oxidase subunit 1 (COI) gene, partial cds; mitochondrial

PP981030.1 Grapholita funebrana voucher NIFoS_DNA_1 cytochrome c oxidase subunit I (COX1) gene, partial cds; mitochondrial

*Hedya nubiferana*

FJ412614.1 Hedya nubiferana voucher UBC-2007-0476 cytochrome oxidase subunit 1 (COI) gene, partial cds; mitochondrial

FJ412615.1 Hedya nubiferana voucher UBC-2007-0475 cytochrome oxidase subunit 1 (COI) gene, partial cds; mitochondrial

FJ412616.1 Hedya nubiferana voucher UBC-2007-0474 cytochrome oxidase subunit 1 (COI) gene, partial cds; mitochondrial

FJ412617.1 Hedya nubiferana voucher UBC-2007-0473 cytochrome oxidase subunit 1 (COI) gene, partial cds; mitochondrial

FJ412618.1 Hedya nubiferana voucher UBC-2007-0255 cytochrome oxidase subunit 1 (COI) gene, partial cds; mitochondrial

FJ412619.1 Hedya nubiferana voucher UBC-2007-0254 cytochrome oxidase subunit 1 (COI) gene, partial cds; mitochondrial

FJ412620.1 Hedya nubiferana voucher UBC-2007-0253 cytochrome oxidase subunit 1 (COI) gene, partial cds; mitochondrial

FJ412621.1 Hedya nubiferana voucher UBC-2007-0252 cytochrome oxidase subunit 1 (COI) gene, partial cds; mitochondrial

FJ412622.1 Hedya nubiferana voucher UBC-2007-0251 cytochrome oxidase subunit 1 (COI) gene, partial cds; mitochondrial

FJ412623.1 Hedya nubiferana voucher UBC-2007-0660 cytochrome oxidase subunit 1 (COI) gene, partial cds; mitochondrial

FJ412624.1 Hedya nubiferana voucher UBC-2007-0079 cytochrome oxidase subunit 1 (COI) gene, partial cds; mitochondrial

FJ412625.1 Hedya nubiferana voucher UBC-2007-0078 cytochrome oxidase subunit 1 (COI) gene, partial cds; mitochondrial

GU707048.1 Hedya nubiferana voucher BC ZSM Lep 25723 cytochrome oxidase subunit 1 (COI) gene, partial cds; mitochondrial

HM391725.1 Hedya nubiferana voucher BC ZSM Lep 23159 cytochrome oxidase subunit 1 (COI) gene, partial cds; mitochondrial

HM391726.1 Hedya nubiferana voucher BC ZSM Lep 23160 cytochrome oxidase subunit 1 (COI) gene, partial cds; mitochondrial

HM391963.1 Hedya nubiferana voucher BC ZSM Lep 28603 cytochrome oxidase subunit 1 (COI) gene, partial cds; mitochondrial

HM875549.1 Hedya nubiferana voucher MM13244 cytochrome oxidase subunit 1 (COI) gene, partial cds; mitochondrial

HM872776.1 Hedya nubiferana voucher MM05336 cytochrome oxidase subunit 1 (COI) gene, partial cds; mitochondrial

HQ563715.1 Hedya nubiferana voucher BC ZSM Lep 38127 cytochrome oxidase subunit 1 (COI) gene, partial cds; mitochondrial

HM871345.1 Hedya nubiferana voucher MM01964 cytochrome oxidase subunit 1 (COI) gene, partial cds; mitochondrial

JF852576.1 Hedya nubiferana voucher BIOUG<CAN_:AVBC 137-10 cytochrome oxidase subunit 1 (COI) gene, partial cds; mitochondrial

JF852589.1 Hedya nubiferana voucher BIOUG<CAN_:AVBC 150-10 cytochrome oxidase subunit 1 (COI) gene, partial cds; mitochondrial

JF852627.1 Hedya nubiferana voucher BIOUG<CAN_:AVBC 189-10 cytochrome oxidase subunit 1 (COI) gene, partial cds; mitochondrial

JF859745.1 Hedya nubiferana voucher TLMF Lep 02161 cytochrome oxidase subunit 1 (COI) gene, partial cds; mitochondrial

KM572600.1 Hedya nubiferana voucher TLMF Lep 09926 cytochrome oxidase subunit 1 (COI) gene, partial cds; mitochondrial

KR446225.1 Hedya nubiferana voucher BIOUG12196-H10 cytochrome oxidase subunit 1 (COI) gene, partial cds; mitochondrial

KX042404.1 Hedya nubiferana voucher TLMF Lep 05213 cytochrome oxidase subunit 1 (COI) gene, partial cds; mitochondrial

KX046088.1 Hedya nubiferana voucher BC ZSM Lep 64305 cytochrome oxidase subunit 1 (COI) gene, partial cds; mitochondrial

KX042898.1 Hedya nubiferana voucher UKLB40E04 cytochrome oxidase subunit 1 (COI) gene, partial cds; mitochondrial

KX048462.1 Hedya nubiferana voucher NHMO-08050 cytochrome oxidase subunit 1 (COI) gene, partial cds; mitochondrial

KX049848.1 Hedya nubiferana voucher RMNH.INS.540675 cytochrome oxidase subunit 1 (COI) gene, partial cds; mitochondrial

KT134485.1 Hedya nubiferana voucher PPBP-2208 cytochrome oxidase subunit 1 (COI) gene, partial cds; mitochondrial

KT136535.1 Hedya nubiferana voucher AVBC 986-11 cytochrome oxidase subunit 1 (COI) gene, partial cds; mitochondrial

KT141436.1 Hedya nubiferana voucher 04HBL006240 cytochrome oxidase subunit 1 (COI) gene, partial cds; mitochondrial

KT144112.1 Hedya nubiferana voucher 04HBL006524 cytochrome oxidase subunit 1 (COI) gene, partial cds; mitochondrial

KT144269.1 Hedya nubiferana voucher 04HBL006257 cytochrome oxidase subunit 1 (COI) gene, partial cds; mitochondrial

KT145308.1 Hedya nubiferana voucher PPBP-1754 cytochrome oxidase subunit 1 (COI) gene, partial cds; mitochondrial

KT147702.1 Hedya nubiferana voucher 04HBL006017 cytochrome oxidase subunit 1 (COI) gene, partial cds; mitochondrial

MG466507.1 Hedya nubiferana voucher BIOUG19151-B02 cytochrome oxidase subunit 1 (COI) gene, partial cds; mitochondrial

OQ563000.1 Hedya nubiferana voucher INV01179 cytochrome oxidase subunit 1 (COI) gene, partial cds; mitochondrial

*Pandemis cerasana*

FJ412859.1 Pandemis cerasana voucher UBC-2007-0635 cytochrome oxidase subunit 1 (COI) gene, partial cds; mitochondrial

FJ412860.1 Pandemis cerasana voucher UBC-2007-0185 cytochrome oxidase subunit 1 (COI) gene, partial cds; mitochondrial

FJ412861.1 Pandemis cerasana voucher UBC-2007-0184 cytochrome oxidase subunit 1 (COI) gene, partial cds; mitochondrial

FJ412862.1 Pandemis cerasana voucher UBC-2007-0183 cytochrome oxidase subunit 1 (COI) gene, partial cds; mitochondrial

FJ412863.1 Pandemis cerasana voucher UBC-2007-0091 cytochrome oxidase subunit 1 (COI) gene, partial cds; mitochondrial

FJ412864.1 Pandemis cerasana voucher UBC-2007-0090 cytochrome oxidase subunit 1 (COI) gene, partial cds; mitochondrial

FJ412865.1 Pandemis cerasana voucher UBC-2007-0089 cytochrome oxidase subunit 1 (COI) gene, partial cds; mitochondrial

FJ412866.1 Pandemis cerasana voucher UBC-2007-0270 cytochrome oxidase subunit 1 (COI) gene, partial cds; mitochondrial

FJ412867.1 Pandemis cerasana voucher UBC-2007-0267 cytochrome oxidase subunit 1 (COI) gene, partial cds; mitochondrial

FJ412868.1 Pandemis cerasana voucher UBC-2007-0266 cytochrome oxidase subunit 1 (COI) gene, partial cds; mitochondrial

GU706514.1 Pandemis cerasana voucher BC ZSM Lep 23132 cytochrome oxidase subunit 1 (COI) gene, partial cds; mitochondrial

GU706516.1 Pandemis cerasana voucher BC ZSM Lep 23131 cytochrome oxidase subunit 1 (COI) gene, partial cds; mitochondrial

GU706686.1 Pandemis cerasana voucher BC ZSM Lep 25140 cytochrome oxidase subunit 1 (COI) gene, partial cds; mitochondrial

GU706690.1 Pandemis cerasana voucher BC ZSM Lep 23410 cytochrome oxidase subunit 1 (COI) gene, partial cds; mitochondrial

GU706692.1 Pandemis cerasana voucher BC ZSM Lep 23411 cytochrome oxidase subunit 1 (COI) gene, partial cds; mitochondrial

GU707209.1 Pandemis cerasana voucher BC ZSM Lep 25552 cytochrome oxidase subunit 1 (COI) gene, partial cds; mitochondrial

HM391896.1 Pandemis cerasana voucher BC ZSM Lep 28480 cytochrome oxidase subunit 1 (COI) gene, partial cds; mitochondrial

HM391898.1 Pandemis cerasana voucher BC ZSM Lep 28483 cytochrome oxidase subunit 1 (COI) gene, partial cds; mitochondrial

HM875528.1 Pandemis cerasana voucher MM13194 cytochrome oxidase subunit 1 (COI) gene, partial cds; mitochondrial

HM871365.1 Pandemis cerasana voucher MM02012 cytochrome oxidase subunit 1 (COI) gene, partial cds; mitochondrial

HM871901.1 Pandemis cerasana voucher MM03256 cytochrome oxidase subunit 1 (COI) gene, partial cds; mitochondrial

JF852599.1 Pandemis cerasana voucher BIOUG<CAN_:AVBC 160-10 cytochrome oxidase subunit 1 (COI) gene, partial cds; mitochondrial

JF852620.1 Pandemis cerasana voucher BIOUG<CAN_:AVBC 182-10 cytochrome oxidase subunit 1 (COI) gene, partial cds; mitochondrial

JF859727.1 Pandemis cerasana voucher TLMF Lep 02143 cytochrome oxidase subunit 1 (COI) gene, partial cds; mitochondrial

JF703081.1 Pandemis cerasana voucher JDDNA4539 cytochrome c oxidase subunit I gene, partial cds; mitochondrial

KM572953.1 Pandemis cerasana voucher TLMF Lep 08018 cytochrome oxidase subunit 1 (COI) gene, partial cds; mitochondrial

KX041165.1 Pandemis cerasana voucher BC ZSM Lep 50727 cytochrome oxidase subunit 1 (COI) gene, partial cds; mitochondrial

KX044035.1 Pandemis cerasana voucher UKLB34D06 cytochrome oxidase subunit 1 (COI) gene, partial cds; mitochondrial

KX047759.1 Pandemis cerasana voucher RMNH.INS.540713 cytochrome oxidase subunit 1 (COI) gene, partial cds; mitochondrial

KX048083.1 Pandemis cerasana voucher RMNH.INS.540866 cytochrome oxidase subunit 1 (COI) gene, partial cds; mitochondrial

KX048773.1 Pandemis cerasana voucher RMNH.INS.540877 cytochrome oxidase subunit 1 (COI) gene, partial cds; mitochondrial

KX049567.1 Pandemis cerasana voucher RMNH.INS.539106 cytochrome oxidase subunit 1 (COI) gene, partial cds; mitochondrial

KT134605.1 Pandemis cerasana voucher AVBC 1010-11 cytochrome oxidase subunit 1 (COI) gene, partial cds; mitochondrial

KT141071.1 Pandemis cerasana voucher AVBC 985-11 cytochrome oxidase subunit 1 (COI) gene, partial cds; mitochondrial

KT144397.1 Pandemis cerasana voucher AVBC 1009-11 cytochrome oxidase subunit 1 (COI) gene, partial cds; mitochondrial

OQ182338.1 Pandemis cerasana voucher TLMF Lep 27759 cytochrome oxidase subunit 1 (COI) gene, partial cds; mitochondrial

OQ563284.1 Pandemis cerasana voucher INV01180 cytochrome oxidase subunit 1 (COI) gene, partial cds; mitochondrial

OR614746.1 Pandemis cerasana voucher WC01671 cytochrome oxidase subunit 1 (COI) gene, partial cds; mitochondrial

OR614747.1 Pandemis cerasana voucher WC02023 cytochrome oxidase subunit 1 (COI) gene, partial cds; mitochondrial

*Pandemis heparana*

FJ412852.1 Pandemis heparana voucher UBC-2007-0478 cytochrome oxidase subunit 1 (COI) gene, partial cds; mitochondrial

FJ412853.1 Pandemis heparana voucher UBC-2007-0941 cytochrome oxidase subunit 1 (COI) gene, partial cds; mitochondrial

FJ412854.1 Pandemis heparana voucher UBC-2007-0639 cytochrome oxidase subunit 1 (COI) gene, partial cds; mitochondrial

FJ412855.1 Pandemis heparana voucher UBC-2007-0638 cytochrome oxidase subunit 1 (COI) gene, partial cds; mitochondrial

FJ412856.1 Pandemis heparana voucher UBC-2007-0637 cytochrome oxidase subunit 1 (COI) gene, partial cds; mitochondrial

FJ412857.1 Pandemis heparana voucher UBC-2007-0824 cytochrome oxidase subunit 1 (COI) gene, partial cds; mitochondrial

FJ412858.1 Pandemis heparana voucher UBC-2007-0268 cytochrome oxidase subunit 1 (COI) gene, partial cds; mitochondrial

GU706683.1 Pandemis heparana voucher BC ZSM Lep 23412 cytochrome oxidase subunit 1 (COI) gene, partial cds; mitochondrial

GU706685.1 Pandemis heparana voucher BC ZSM Lep 25139 cytochrome oxidase subunit 1 (COI) gene, partial cds; mitochondrial

HM875114.1 Pandemis heparana voucher MM11856 cytochrome oxidase subunit 1 (COI) gene, partial cds; mitochondrial

HM875529.1 Pandemis heparana voucher MM13196 cytochrome oxidase subunit 1 (COI) gene, partial cds; mitochondrial

HM902006.1 Lepidoptera sp. BOLD:AAA9254 voucher BC ZSM Lep 23134 cytochrome oxidase subunit 1 (COI) gene, partial cds; mitochondrial

HM871366.1 Pandemis heparana voucher MM02014 cytochrome oxidase subunit 1 (COI) gene, partial cds; mitochondrial

JF703085.1 Pandemis heparana voucher JDDNA4590 cytochrome c oxidase subunit I gene, partial cds; mitochondrial

KF491974.1 Pandemis heparana voucher AYK-04-5721 cytochrome oxidase subunit 1 (COI) gene, partial cds; mitochondrial

KM544704.1 Pandemis heparana voucher BIOUG07289-D08 cytochrome oxidase subunit 1 (COI) gene, partial cds; mitochondrial

KM545578.1 Pandemis heparana voucher BIOUG07289-E04 cytochrome oxidase subunit 1 (COI) gene, partial cds; mitochondrial

KM550940.1 Pandemis heparana voucher BIOUG06978-C04 cytochrome oxidase subunit 1 (COI) gene, partial cds; mitochondrial

KX042973.1 Pandemis heparana voucher UKLB5B05 cytochrome oxidase subunit 1 (COI) gene, partial cds; mitochondrial

KX043162.1 Pandemis heparana voucher UKLB40B12 cytochrome oxidase subunit 1 (COI) gene, partial cds; mitochondrial

KX043674.1 Pandemis heparana voucher UKLB38G12 cytochrome oxidase subunit 1 (COI) gene, partial cds; mitochondrial

KX044028.1 Pandemis heparana voucher UKLB38H04 cytochrome oxidase subunit 1 (COI) gene, partial cds; mitochondrial

KX048073.1 Pandemis heparana voucher RMNH.INS.539088 cytochrome oxidase subunit 1 (COI) gene, partial cds; mitochondrial

KX048140.1 Pandemis heparana voucher RMNH.INS.538490 cytochrome oxidase subunit 1 (COI) gene, partial cds; mitochondrial

KX048274.1 Pandemis heparana voucher RMNH.INS.539085 cytochrome oxidase subunit 1 (COI) gene, partial cds; mitochondrial

KX049030.1 Pandemis heparana voucher NHMO-08212 cytochrome oxidase subunit 1 (COI) gene, partial cds; mitochondrial

KX049077.1 Pandemis heparana voucher RMNH.INS.539063 cytochrome oxidase subunit 1 (COI) gene, partial cds; mitochondrial

KX049338.1 Pandemis heparana voucher RMNH.INS.539087 cytochrome oxidase subunit 1 (COI) gene, partial cds; mitochondrial

KX049555.1 Pandemis heparana voucher RMNH.INS.544485 cytochrome oxidase subunit 1 (COI) gene, partial cds; mitochondrial

KX050085.1 Pandemis heparana voucher RMNH.INS.539098 cytochrome oxidase subunit 1 (COI) gene, partial cds; mitochondrial

KX045737.1 Pandemis heparana voucher BC ZSM Lep 64378 cytochrome oxidase subunit 1 (COI) gene, partial cds; mitochondrial

MF049731.1 Pandemis heparana isolate DLS110709.107 cytochrome oxidase subunit I (COI) gene, partial cds; mitochondrial

MZ610464.1 Pandemis heparana voucher MM24753 cytochrome oxidase subunit 1 (COI) gene, partial cds; mitochondrial

OQ181823.1 Pandemis heparana voucher TLMF Lep 29499 cytochrome oxidase subunit 1 (COI) gene, partial cds; mitochondrial

OQ562250.1 Pandemis heparana voucher INV03250 cytochrome oxidase subunit 1 (COI) gene, partial cds; mitochondrial

OQ563231.1 Pandemis heparana voucher INV03139 cytochrome oxidase subunit 1 (COI) gene, partial cds; mitochondrial

OQ563935.1 Pandemis heparana voucher INV08624 cytochrome oxidase subunit 1 (COI) gene, partial cds; mitochondrial

OQ564256.1 Pandemis heparana voucher INV03333 cytochrome oxidase subunit 1 (COI) gene, partial cds; mitochondrial

Pandemis pyrusana

JF703088.1 Pandemis pyrusana voucher JDDNA4551 cytochrome c oxidase subunit I gene, partial cds; mitochondrial

KM545241.1 Pandemis pyrusana voucher 10BBCLP-2502 cytochrome oxidase subunit 1 (COI) gene, partial cds; mitochondrial

*Spilonota lechriaspis*

MN565346.1 Spilonota lechriaspis isolate 20160123 cytochrome oxidase subunit I (COI) gene, partial cds; mitochondrial

MN565347.1 Spilonota lechriaspis isolate 20160124 cytochrome oxidase subunit I (COI) gene, partial cds; mitochondrial

MN565348.1 Spilonota lechriaspis isolate 20160125 cytochrome oxidase subunit I (COI) gene, partial cds; mitochondrial

MN565349.1 Spilonota lechriaspis isolate 20160126 cytochrome oxidase subunit I (COI) gene, partial cds; mitochondrial

MN565350.1 Spilonota lechriaspis isolate 20160127 cytochrome oxidase subunit I (COI) gene, partial cds; mitochondrial

MN565351.1 Spilonota lechriaspis isolate 20160128 cytochrome oxidase subunit I (COI) gene, partial cds; mitochondrial

MN565352.1 Spilonota lechriaspis isolate 20160129 cytochrome oxidase subunit I (COI) gene, partial cds; mitochondrial

MN565353.1 Spilonota lechriaspis isolate 20160130 cytochrome oxidase subunit I (COI) gene, partial cds; mitochondrial

MN565354.1 Spilonota lechriaspis isolate 20160131 cytochrome oxidase subunit I (COI) gene, partial cds; mitochondrial

MN565355.1 Spilonota lechriaspis isolate 20160132 cytochrome oxidase subunit I (COI) gene, partial cds; mitochondrial

MN565356.1 Spilonota lechriaspis isolate 20160133 cytochrome oxidase subunit I (COI) gene, partial cds; mitochondrial

MN565357.1 Spilonota lechriaspis isolate 20160134 cytochrome oxidase subunit I (COI) gene, partial cds; mitochondrial

MN565358.1 Spilonota lechriaspis isolate 20160135 cytochrome oxidase subunit I (COI) gene, partial cds; mitochondrial

MN565359.1 Spilonota lechriaspis isolate 20160136 cytochrome oxidase subunit I (COI) gene, partial cds; mitochondrial

MN565360.1 Spilonota lechriaspis isolate 20160137 cytochrome oxidase subunit I (COI) gene, partial cds; mitochondrial

MN565361.1 Spilonota lechriaspis isolate 20160138 cytochrome oxidase subunit I (COI) gene, partial cds; mitochondrial

MN565362.1 Spilonota lechriaspis isolate 20160139 cytochrome oxidase subunit I (COI) gene, partial cds; mitochondrial

*Archips podanus*

GU706520.1 Archips podanus voucher BC ZSM Lep 23123 cytochrome oxidase subunit 1 (COI) gene, partial cds; mitochondrial

GU706918.1 Archips podanus voucher BC ZSM Lep 23398 cytochrome oxidase subunit 1 (COI) gene, partial cds; mitochondrial

GU706921.1 Archips podanus voucher BC ZSM Lep 23399 cytochrome oxidase subunit 1 (COI) gene, partial cds; mitochondrial

GU989458.1 Archips podanus cytochrome oxidase subunit I (COI) gene, partial cds; mitochondrial

HM387238.1 Archips podanus voucher KB08024 cytochrome oxidase subunit 1 (COI) gene, partial cds; mitochondrial

HM876440.1 Archips podanus voucher MM15652 cytochrome oxidase subunit 1 (COI) gene, partial cds; mitochondrial

HM876441.1 Archips podanus voucher MM15653 cytochrome oxidase subunit 1 (COI) gene, partial cds; mitochondrial

HM872723.1 Archips podanus voucher MM05228 cytochrome oxidase subunit 1 (COI) gene, partial cds; mitochondrial

HM873440.1 Archips podanus voucher MM06722 cytochrome oxidase subunit 1 (COI) gene, partial cds; mitochondrial

HQ955214.1 Archips podanus voucher BC ZSM Lep 25563 cytochrome oxidase subunit 1 (COI) gene, partial cds; mitochondrial

JF859739.1 Archips podana voucher TLMF Lep 02155 cytochrome oxidase subunit 1 (COI) gene, partial cds; mitochondrial

JN286435.1 Archips podanus voucher MM15190 cytochrome oxidase subunit 1 (COI) gene, partial cds; mitochondrial

JF703026.1 Archips podana voucher JDDNA4557 cytochrome c oxidase subunit I gene, partial cds; mitochondrial

KM573356.1 Archips podana voucher TLMF Lep 08569 cytochrome oxidase subunit 1 (COI) gene, partial cds; mitochondrial

KX044044.1 Archips podanus voucher UKLB40C02 cytochrome oxidase subunit 1 (COI) gene, partial cds; mitochondrial

KX047738.1 Archips podanus voucher NHMO-08179 cytochrome oxidase subunit 1 (COI) gene, partial cds; mitochondrial

KX048723.1 Archips podanus voucher RMNH.INS.540848 cytochrome oxidase subunit 1 (COI) gene, partial cds; mitochondrial

KX049119.1 Archips podanus voucher RMNH.INS.540974 cytochrome oxidase subunit 1 (COI) gene, partial cds; mitochondrial

KX440876.1 Archips podanus isolate SQ0926114 cytochrome c oxidase subunit I (COI) gene, partial cds; mitochondrial

MF049916.1 Archips podanus isolate DLS110710.121 cytochrome oxidase subunit I (COI) gene, partial cds; mitochondrial

MF050403.1 Archips podanus isolate JG100802.042 cytochrome oxidase subunit I (COI) gene, partial cds; mitochondrial

MF055474.1 Archips podanus isolate WLS130803.543 cytochrome oxidase subunit I (COI) gene, partial cds; mitochondrial

MN131238.1 Archips podanus isolate Lep0098 cytochrome oxidase subunit 1 (COI) gene, partial cds; mitochondrial

MN132044.1 Archips podanus isolate Lep3377 cytochrome oxidase subunit 1 (COI) gene, partial cds; mitochondrial

OQ181942.1 Archips podanus voucher TLMF Lep 29497 cytochrome oxidase subunit 1 (COI) gene, partial cds; mitochondrial

OQ181985.1 Archips podanus voucher TLMF Lep 32782 cytochrome oxidase subunit 1 (COI) gene, partial cds; mitochondrial

*Archips rosana*

GU706919.1 Archips rosana voucher BC ZSM Lep 23402 cytochrome oxidase subunit 1 (COI) gene, partial cds; mitochondrial

GU707055.1 Archips rosana voucher BC ZSM Lep 25709 cytochrome oxidase subunit 1 (COI) gene, partial cds; mitochondrial

HM874607.1 Archips rosana voucher MM10167 cytochrome oxidase subunit 1 (COI) gene, partial cds; mitochondrial

HM902002.1 Lepidoptera sp. BOLD:AAB9404 voucher BC ZSM Lep 23125 cytochrome oxidase subunit 1 (COI) gene, partial cds; mitochondrial

HM871367.1 Archips rosana voucher MM02016 cytochrome oxidase subunit 1 (COI) gene, partial cds; mitochondrial

JF854249.1 Archips rosana voucher MM18256 cytochrome oxidase subunit 1 (COI) gene, partial cds; mitochondrial

JF703028.1 Archips rosana voucher JDDNA4563 cytochrome c oxidase subunit I gene, partial cds; mitochondrial

KM572636.1 Archips rosana voucher TLMF Lep 08029 cytochrome oxidase subunit 1 (COI) gene, partial cds; mitochondrial

KM573536.1 Archips rosana voucher TLMF Lep 08262 cytochrome oxidase subunit 1 (COI) gene, partial cds; mitochondrial

KX046387.1 Archips rosana voucher BC ZSM Lep 64380 cytochrome oxidase subunit 1 (COI) gene, partial cds; mitochondrial

KX046736.1 Archips rosana voucher BC ZSM Lep add 0045 cytochrome oxidase subunit 1 (COI) gene, partial cds; mitochondrial

KX045476.1 Archips rosana voucher BC ZSM Lep 64302 cytochrome oxidase subunit 1 (COI) gene, partial cds; mitochondrial

KU874198.1 Archips rosana voucher UAM:Ento:129783 cytochrome oxidase subunit 1 (COI) gene, partial cds; mitochondrial

MZ607857.1 Archips rosana voucher MM24348 cytochrome oxidase subunit 1 (COI) gene, partial cds; mitochondrial

OR352908.1 Archips rosana isolate ar23 cytochrome c oxidase subunit I (COX1) gene, partial cds; mitochondrial

OQ182931.1 Archips rosana voucher KLM Lep 14903 cytochrome oxidase subunit 1 (COI) gene, partial cds; mitochondrial

OQ563464.1 Archips rosana voucher INV03160 cytochrome oxidase subunit 1 (COI) gene, partial cds; mitochondrial

OQ564212.1 Archips rosana voucher INV00879 cytochrome oxidase subunit 1 (COI) gene, partial cds; mitochondrial

*Archips xylosteana*

GU706917.1 Archips xylosteana voucher BC ZSM Lep 25141 cytochrome oxidase subunit 1 (COI) gene, partial cds; mitochondrial

HM387018.1 Archips xylosteana voucher MM09757 cytochrome oxidase subunit 1 (COI) gene, partial cds; mitochondrial

HM405736.1 Archips xylosteana voucher UKLB28C07 cytochrome oxidase subunit 1 (COI) gene, partial cds; mitochondrial

HM872630.1 Archips xylosteana voucher MM04987 cytochrome oxidase subunit 1 (COI) gene, partial cds; mitochondrial

HM874308.1 Archips xylosteana voucher MM09490 cytochrome oxidase subunit 1 (COI) gene, partial cds; mitochondrial

HQ955217.1 Archips xylosteana voucher BC ZSM Lep 25566 cytochrome oxidase subunit 1 (COI) gene, partial cds; mitochondrial

JF859738.1 Archips xylosteana voucher TLMF Lep 02154 cytochrome oxidase subunit 1 (COI) gene, partial cds; mitochondrial

JF859742.1 Archips xylosteana voucher TLMF Lep 02158 cytochrome oxidase subunit 1 (COI) gene, partial cds; mitochondrial

JF703030.1 Archips xylosteana voucher JDDNA4559 cytochrome c oxidase subunit I gene, partial cds; mitochondrial

KM572080.1 Archips xylosteana voucher TLMF Lep 08013 cytochrome oxidase subunit 1 (COI) gene, partial cds; mitochondrial

KX040156.1 Archips xylosteana voucher BC ZSM Lep 50720 cytochrome oxidase subunit 1 (COI) gene, partial cds; mitochondrial

KX043191.1 Archips xylosteana voucher UKLB38B07 cytochrome oxidase subunit 1 (COI) gene, partial cds; mitochondrial

KX047972.1 Archips xylosteana voucher RMNH.INS.540875 cytochrome oxidase subunit 1 (COI) gene, partial cds; mitochondrial

KX048038.1 Archips xylosteana voucher RMNH.INS.540838 cytochrome oxidase subunit 1 (COI) gene, partial cds; mitochondrial

KX048350.1 Archips xylosteana voucher RMNH.INS.540876 cytochrome oxidase subunit 1 (COI) gene, partial cds; mitochondrial

KX048585.1 Archips xylosteana voucher RMNH.INS.540834 cytochrome oxidase subunit 1 (COI) gene, partial cds; mitochondrial

KX049227.1 Archips xylosteana voucher RMNH.INS.540899 cytochrome oxidase subunit 1 (COI) gene, partial cds; mitochondrial

KX049432.1 Archips xylosteana voucher RMNH.INS.540853 cytochrome oxidase subunit 1 (COI) gene, partial cds; mitochondrial

KX049445.1 Archips xylosteana voucher RMNH.INS.540849 cytochrome oxidase subunit 1 (COI) gene, partial cds; mitochondrial

KX044296.1 Archips xylosteana voucher BC ZSM Lep 61218 cytochrome oxidase subunit 1 (COI) gene, partial cds; mitochondrial

KX045520.1 Archips xylosteana voucher BC ZSM Lep 64350 cytochrome oxidase subunit 1 (COI) gene, partial cds; mitochondrial

MF050572.1 Archips xylosteana isolate JG100812.380 cytochrome oxidase subunit I (COI) gene, partial cds; mitochondrial

*Grapholita molesta*

HQ538466.1_Grapholita_molesta_cytochrome_oxidase_subunit_I_(COI)_gene,_partial_cds;_mitochondrial

GU096464.1 Grapholita molesta voucher jflandry1668 cytochrome oxidase subunit 1 (COI) gene, partial cds; mitochondrial

GU096466.1 Grapholita molesta voucher jflandry1667 cytochrome oxidase subunit 1 (COI) gene, partial cds; mitochondrial

GU096467.1 Grapholita molesta voucher jflandry1666 cytochrome oxidase subunit 1 (COI) gene, partial cds; mitochondrial

GU096468.1 Grapholita molesta voucher jflandry1669 cytochrome oxidase subunit 1 (COI) gene, partial cds; mitochondrial

HQ538466.1 Grapholita molesta cytochrome oxidase subunit I (COI) gene, partial cds; mitochondrial

JN305849.1 Lepidoptera sp. BOLD:AAB0523 voucher BIOUG<CAN_:MDOK-4264 cytochrome oxidase subunit 1 (COI) gene, partial cds; mitochondrial

JF733841.1 Grapholita molesta cytochrome oxidase subunit I (COI) gene, partial cds; mitochondrial

KC136080.1 Grapholita molesta voucher soonR4 cytochrome oxidase subunit I (COI) gene, partial cds; mitochondrial

KC136081.1 Grapholita molesta voucher soonR3 cytochrome oxidase subunit I (COI) gene, partial cds; mitochondrial

KC136082.1 Grapholita molesta voucher soon112 cytochrome oxidase subunit I (COI) gene, partial cds; mitochondrial

KC136083.1 Grapholita molesta voucher soon3 cytochrome oxidase subunit I (COI) gene, partial cds; mitochondrial

KC136084.1 Grapholita molesta voucher soon2 cytochrome oxidase subunit I (COI) gene, partial cds; mitochondrial

KC136085.1 Grapholita molesta voucher soon4 cytochrome oxidase subunit I (COI) gene, partial cds; mitochondrial

KC136086.1 Grapholita molesta voucher soon5 cytochrome oxidase subunit I (COI) gene, partial cds; mitochondrial

KC136087.1 Grapholita molesta voucher soon21 cytochrome oxidase subunit I (COI) gene, partial cds; mitochondrial

KC136088.1 Grapholita molesta voucher soon23 cytochrome oxidase subunit I (COI) gene, partial cds; mitochondrial

KC136089.1 Grapholita molesta voucher soon24 cytochrome oxidase subunit I (COI) gene, partial cds; mitochondrial

KC136090.1 Grapholita molesta voucher soon25 cytochrome oxidase subunit I (COI) gene, partial cds; mitochondrial

KF396799.1 Grapholita molesta voucher 11ANIC-12980 cytochrome oxidase subunit 1 (COI) gene, partial cds; mitochondrial

KF405474.1 Grapholita molesta voucher 11ANIC-12979 cytochrome oxidase subunit 1 (COI) gene, partial cds; mitochondrial

KJ027508.1 Grapholita molesta voucher HEAIQ 20130902 cytochrome oxidase subunit I (COI) gene, partial cds; mitochondrial

KJ833910.1 Grapholita molesta voucher lxy3110134 cytochrome oxidase subunit I (COI) gene, partial cds; mitochondrial

KJ833911.1 Grapholita molesta voucher lxy3110135 cytochrome oxidase subunit I (COI) gene, partial cds; mitochondrial

KJ833912.1 Grapholita molesta voucher lxy3110136 cytochrome oxidase subunit I (COI) gene, partial cds; mitochondrial

KJ833913.1 Grapholita molesta voucher lxy3110137 cytochrome oxidase subunit I (COI) gene, partial cds; mitochondrial

KJ833914.1 Grapholita molesta voucher lxy3110138 cytochrome oxidase subunit I (COI) gene, partial cds; mitochondrial

KJ833915.1 Grapholita molesta voucher lxy3110139 cytochrome oxidase subunit I (COI) gene, partial cds; mitochondrial

KJ833916.1 Grapholita molesta voucher lxy3110140 cytochrome oxidase subunit I (COI) gene, partial cds; mitochondrial

KJ833917.1 Grapholita molesta voucher lxy3110141 cytochrome oxidase subunit I (COI) gene, partial cds; mitochondrial

KJ833918.1 Grapholita molesta voucher lxy3110142 cytochrome oxidase subunit I (COI) gene, partial cds; mitochondrial

KJ833919.1 Grapholita molesta voucher lxy3110143 cytochrome oxidase subunit I (COI) gene, partial cds; mitochondrial

KJ833920.1 Grapholita molesta voucher lxy3110144 cytochrome oxidase subunit I (COI) gene, partial cds; mitochondrial

KJ833921.1 Grapholita molesta voucher lxy3110145 cytochrome oxidase subunit I (COI) gene, partial cds; mitochondrial

KJ833922.1 Grapholita molesta voucher lxy3110146 cytochrome oxidase subunit I (COI) gene, partial cds; mitochondrial

KJ833923.1 Grapholita molesta voucher lxy3110147 cytochrome oxidase subunit I (COI) gene, partial cds; mitochondrial

KJ833924.1 Grapholita molesta voucher lxy3110148 cytochrome oxidase subunit I (COI) gene, partial cds; mitochondrial

KJ833925.1 Grapholita molesta voucher lxy3110149 cytochrome oxidase subunit I (COI) gene, partial cds; mitochondrial

KJ833926.1 Grapholita molesta voucher lxy3110150 cytochrome oxidase subunit I (COI) gene, partial cds; mitochondrial

KJ833927.1 Grapholita molesta voucher lxy3110151 cytochrome oxidase subunit I (COI) gene, partial cds; mitochondrial

KJ833928.1 Grapholita molesta voucher lxy3110152 cytochrome oxidase subunit I (COI) gene, partial cds; mitochondrial

KJ833929.1 Grapholita molesta voucher lxy3110153 cytochrome oxidase subunit I (COI) gene, partial cds; mitochondrial

KJ833930.1 Grapholita molesta voucher lxy3110154 cytochrome oxidase subunit I (COI) gene, partial cds; mitochondrial

KJ833931.1 Grapholita molesta voucher lxy3110155 cytochrome oxidase subunit I (COI) gene, partial cds; mitochondrial

KJ833932.1 Grapholita molesta voucher lxy3110156 cytochrome oxidase subunit I (COI) gene, partial cds; mitochondrial

KJ833933.1 Grapholita molesta voucher lxy3110157 cytochrome oxidase subunit I (COI) gene, partial cds; mitochondrial

KJ833934.1 Grapholita molesta voucher lxy3110158 cytochrome oxidase subunit I (COI) gene, partial cds; mitochondrial

KJ833935.1 Grapholita molesta voucher lxy3110159 cytochrome oxidase subunit I (COI) gene, partial cds; mitochondrial

KJ833936.1 Grapholita molesta voucher lxy3110160 cytochrome oxidase subunit I (COI) gene, partial cds; mitochondrial

KJ833937.1 Grapholita molesta voucher lxy3110161 cytochrome oxidase subunit I (COI) gene, partial cds; mitochondrial

KJ833938.1 Grapholita molesta voucher lxy3110162 cytochrome oxidase subunit I (COI) gene, partial cds; mitochondrial

KJ833939.1 Grapholita molesta voucher lxy3110163 cytochrome oxidase subunit I (COI) gene, partial cds; mitochondrial

KP072625.1 Grapholita molesta voucher Ev-06-1108 cytochrome oxidase subunit I (COI) gene, partial cds; mitochondrial

KP072626.1 Grapholita molesta voucher Ev-06-1247 cytochrome oxidase subunit I (COI) gene, partial cds; mitochondrial

KP072627.1 Grapholita molesta voucher Ev-06-811 cytochrome oxidase subunit I (COI) gene, partial cds; mitochondrial

KP072628.1 Grapholita molesta voucher Ev-06-2183 cytochrome oxidase subunit I (COI) gene, partial cds; mitochondrial

KP072629.1 Grapholita molesta voucher Ev-07-4041 cytochrome oxidase subunit I (COI) gene, partial cds; mitochondrial

KP072630.1 Grapholita molesta voucher Ev-07-4634 cytochrome oxidase subunit I (COI) gene, partial cds; mitochondrial

KP072631.1 Grapholita molesta voucher Ev-07-4680 cytochrome oxidase subunit I (COI) gene, partial cds; mitochondrial

KP072632.1 Grapholita molesta voucher Ev-07-4840 cytochrome oxidase subunit I (COI) gene, partial cds; mitochondrial

KP072633.1 Grapholita molesta voucher Ev-07-5292 cytochrome oxidase subunit I (COI) gene, partial cds; mitochondrial

KP072634.1 Grapholita molesta voucher Ev-07-4841 cytochrome oxidase subunit I (COI) gene, partial cds; mitochondrial

KP072635.1 Grapholita molesta voucher Ev-07-5314 cytochrome oxidase subunit I (COI) gene, partial cds; mitochondrial

KP072636.1 Grapholita molesta voucher Ev-07-5409 cytochrome oxidase subunit I (COI) gene, partial cds; mitochondrial

KP072637.1 Grapholita molesta voucher Ev-07-5592 cytochrome oxidase subunit I (COI) gene, partial cds; mitochondrial

KP072638.1 Grapholita molesta voucher Ev-10-5502 cytochrome oxidase subunit I (COI) gene, partial cds; mitochondrial

KP072639.1 Grapholita molesta voucher Ev-10-5503 cytochrome oxidase subunit I (COI) gene, partial cds; mitochondrial

KP072640.1 Grapholita molesta voucher Ev-10-5846 cytochrome oxidase subunit I (COI) gene, partial cds; mitochondrial

KP072641.1 Grapholita molesta voucher Ev-10-5331 cytochrome oxidase subunit I (COI) gene, partial cds; mitochondrial

KX377426.1 Grapholita molesta haplotype Hap_1 cytochrome oxidase subunit I (COI) gene, partial cds; mitochondrial

KX377427.1 Grapholita molesta haplotype Hap_2 cytochrome oxidase subunit I (COI) gene, partial cds; mitochondrial

KX377428.1 Grapholita molesta haplotype Hap_3 cytochrome oxidase subunit I (COI) gene, partial cds; mitochondrial

KX377429.1 Grapholita molesta haplotype Hap_4 cytochrome oxidase subunit I (COI) gene, partial cds; mitochondrial

KX377430.1 Grapholita molesta haplotype Hap_5 cytochrome oxidase subunit I (COI) gene, partial cds; mitochondrial

MN565340.1 Grapholita molesta isolate 20160117 cytochrome oxidase subunit I (COI) gene, partial cds; mitochondrial

LC672548.1 Grapholita molesta mitochondrial COI gene for cytochrome oxidase subunit I, partial cds

OP970231.1 Grapholita molesta voucher HNU_DNA_3564 cytochrome c oxidase subunit I (COX1) gene, partial cds; mitochondrial

OP970232.1 Grapholita molesta voucher HNU_DNA_5471 cytochrome c oxidase subunit I (COX1) gene, partial cds; mitochondrial

OQ836369.1 Grapholita molesta voucher JWB-09-0045 cytochrome c oxidase subunit I (COX1) gene, partial cds; mitochondrial

PX380964.1 Grapholita molesta voucher CSCA_25C089 cytochrome c oxidase subunit I (COX1) gene, partial cds; mitochondrial

PZ017143.1 Grapholita molesta voucher HNU_DNA_11762 cytochrome c oxidase subunit I (COX1) gene, partial cds; mitochondrial

PZ017145.1 Grapholita molesta voucher HNU_DNA_11778 cytochrome c oxidase subunit I (COX1) gene, partial cds; mitochondrial

PZ017147.1 Grapholita molesta voucher HNU_DNA_11955 cytochrome c oxidase subunit I (COX1) gene, partial cds; mitochondrial

PZ017148.1 Grapholita molesta voucher HNU_DNA_11960 cytochrome c oxidase subunit I (COX1) gene, partial cds; mitochondrial

PZ018919.1 Grapholita molesta voucher HNU_DNA_11441 cytochrome c oxidase subunit I (COX1) gene, partial cds; mitochondrial

PZ018921.1 Grapholita molesta voucher HNU_DNA_11766 cytochrome c oxidase subunit I (COX1) gene, partial cds; mitochondrial

PZ018923.1 Grapholita molesta voucher HNU_DNA_11961 cytochrome c oxidase subunit I (COX1) gene, partial cds; mitochondrial

PZ018924.1 Grapholita molesta voucher HNU_DNA_11944 cytochrome c oxidase subunit I (COX1) gene, partial cds; mitochondrial

PZ018925.1 Grapholita molesta voucher HNU_DNA_11970 cytochrome c oxidase subunit I (COX1) gene, partial cds; mitochondrial
